# Citizen science reveals host‐switching in louse flies and keds (Diptera: Hippoboscidae) during a period of anthropogenic change

**DOI:** 10.1111/mve.70029

**Published:** 2025-11-01

**Authors:** Denise C. Wawman, Adrian L. Smith, Ben C. Sheldon

**Affiliations:** ^1^ Edward Grey Institute of Field Ornithology, Department of Biology, Life and Mind Building University of Oxford Oxford UK; ^2^ Department of Biology, Life and Mind Building University of Oxford Oxford UK

**Keywords:** birds, citizen science, climate change, host–parasite interactions, vectors, wildlife disease

## Abstract

The Hippoboscidae (Diptera) are a family of obligate blood‐feeding ectoparasites of birds (louse flies) and mammals (keds) that are known to vector pathogenic agents. Citizen scientists collected 4365 hippoboscids of 12 species, from 117 host species, in the UK, Ireland and the Isle of Man, as part of the ‘Mapping the UK's Flat Flies Project’. Of the 212 host—parasite interactions recorded, 70 were previously unreported in the region. Analyses of host characteristics showed evidence of niche separation by host size of the sympatric generalist species *Ornithomya avicularia* (L.) and *Ornithomya fringillina* (Curtis). Comparisons with data from a previous study, published in 1962, showed that all three generalist species in the genus *Ornithomya* increased their host associations during a period of climate and other anthropogenic changes: for example, the switch by some species of gulls (Laridae) to anthropogenic food sources has occurred over the same period that louse flies have started to parasitize them. These changes may have consequences for human and other animal health.

## Graphical abstract

- A study of louse flies in the United Kingdom, Isle of Man, and Ireland found 212 different interactions between Hippoboscidae and their hosts, of which 70 were previously unrecorded.
- No louse flies were found on aquatic species of birds.
- Host‐switching to gulls (Laridae) has occurred during a period in which these species have started relying on anthropogenic food sources and less on marine food sources.

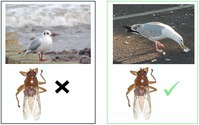

## INTRODUCTION

Humans *Homo sapiens* (L.) (Hominidae: Homininae) have been making major changes to their environment since they discovered fire in the Early Pleistocene Epoch, but these changes have intensified over the millennia, as humans started farming, increased their population and developed industrial processes (Lewis & Maslin, 2015). Anthropogenic changes in land use, with the consequent decrease in the area of habitat available for other species, species introductions, competition for resources, and the exploitation of human resources, such as crops, food provided for wildlife and human waste, bring species into contact that may not have previously shared habitat (Gordon, 2018; Hanmer et al., 2022; O'Hanlon et al., 2025; Preininger et al., 2019; Shutt & Lees, 2021; Spelt et al., 2019). Burning fossil fuels has raised atmospheric carbon dioxide and increased the global mean temperature, with the rise in temperature accelerating rapidly from the mid‐1980s (Hartmann et al., 2013).

It has been predicted that, with climate change, parasites are more likely to switch to novel host species, in association with range shifts and changes in host availability (Brooks & Hoberg, 2007). Migrant birds are known to transport parasites between their summer breeding grounds, stop‐over sites and over‐wintering areas (Burnus et al., 2025) further increasing the potential for avian parasites to establish on new hosts in new regions. Specialisation by parasites may limit possible host interactions and parasite fitness if they are not on their preferred host, due to the host's defence mechanisms, whether these are behavioural, mechanical or immunological (Møller et al., 2005). However, some species of parasites may be able to survive for several generations on sub‐optimal hosts (Araujo et al., 2015).

The Hippoboscidae (Diptera) are a family of obligate ectoparasites of birds (louse flies or flat flies) and mammals (keds) that feed on their hosts' blood. There are over 200 species described worldwide (Dick, 2018), of which 15 have occurred naturally in the United Kingdom (UK), Ireland and the Isle of Man (collectively referred to hereafter as ‘the region’) (Chandler, 2024). An additional two species have been accidentally imported but have not become established (Chandler, 2024). Of the naturally occurring species, 11 are known to breed on hosts in the region. Three species of keds are found on mammals: *Hippobosca equina* L., *Lipoptena cervi* (L.), and *Melophagus ovinus* (L.). There are eight breeding species of louse flies on birds in the region: *Crataerina pallida* (Latreille), *Ornithomya avicularia* (L.), *Ornithomya biloba* (Dufour), *Ornithomya chloropus* (Bergroth), *Ornithomya fringillina* (Curtis), *Pseudolynchia canariensis* (Macquart), *Pseudolynchia garzettae* (Rondani), and *Stenepteryx hirundinis* (L.) (Hutson, 1984; Wawman, 2024).

Some species of hippoboscid are generalists, able to feed and breed successfully on a range of hosts, but others are monoxenous or stenoxenous, restricted to either a single host or a few host species (Hutson, 1984). *Ornithomya avicularia*, *O. chloropus*, and *O. fringillina* are generalists (Corbet, 1956; Hill, 1962a), and individual *O. chloropus* are known to move between host species (Corbet, 1956), whereas the other species in the region have more restricted host ranges (Hutson, 1984; Wawman, 2024).

Host‐switching has occurred on multiple occasions throughout the evolutionary history of the Hippoboscidae, including switches between birds and mammals (de Moya, 2019; Petersen et al., 2007) and the occurrence of mammalian *Babesia* spp. (Starcovici) (Piroplasmida: Babesiidae) in *Ornithomya*, which normally parasitize birds, is likely to be due to host‐switching (Čisovská Bazsalovicsová et al., 2023). Substantial range shifts have occurred in the generalist *Ornithomya* species (Wawman, 2025a), and new species have colonized the region (Wawman, 2024), increasing the potential for host switching.

The Hippoboscidae are known to vector a range of diseases, and a large range of pathogens has been identified from them (Bezerra‐Santos & Otranto, 2020); thus, host‐switching may have implications for both human and other animal health. Some of these pathogens are known to be zoonotic; for example, *Icosta americana* (Leach) (Diptera: Hippoboscidae) has been shown to harbour West Nile Virus (WNV) *Orthoflavivirus nilemse* (Smithburn et al.) (Amarillovirales: Flaviviridae) (Farajollahi et al., 2005). The bacterium, *Bartonella schoenbuchensis*, Dehio et al. (Hyphomicrobiales: Bartonellaceae), which causes deer ked dermatitis in humans, has been shown to be vertically transmitted in *L. cervi* (de Bruin et al., 2015).

While a range of hosts have been reported for some species of hippoboscids, there have been few large‐scale studies of their host species distributions, covering a wide range of hosts and ectoparasites. In the UK, the only in‐depth study from the region was published in 1962, using records from museums and published literature, when analytical techniques and data visualisation were limited by a lack of computing power, and it only covers three species in the genus *Ornithomya* (Hill, 1962a). More recent systematic studies of host distribution restricted themselves to either louse flies (Keve et al., 2024; Lee et al., 2022; Lehikoinen et al., 2021; Levesque‐Beaudin & Sinclair, 2021; Nartshuk et al., 2022) or keds (Salvetti et al., 2020), the exception being a study of the common Hippoboscidae of Slovakia (Mlynárová et al., 2024).

This study uses data obtained from a large citizen science project, the Mapping the UK's Flat Flies Project, to explore the host—parasite relationships of both keds and louse flies in the UK, Ireland and Isle of Man. It aims to determine the current host—parasite relationships and to compare the new data for the generalist *Ornithomya* species with that collected for the 1962 study (Hill, 1962a) to look for evidence of host switching, which has been predicted to occur with climate change, and to produce an up‐to‐date list of Hippobosid‐host interactions for the region.

## MATERIALS AND METHODS

The methods of collection and identification of flies have been previously described (Wawman, 2024) and are only described briefly here for completeness. From 2020 onwards, British Trust for Ornithology (BTO) bird ringers were recruited to collect flat flies that left birds during normal bird ringing activities. Ringers known to be catching less frequently ringed groups of birds were approached directly to look for louse flies on wildfowl, seabirds, waders, herons and egrets, gulls and terns, birds of prey, nightjars etc. All UK bird observatories were also invited to take part. Ringers were sent a collection kit and asked to return any flies that they had collected at the end of the season with their associated metadata, including date, location and host species.

A small number of flies was sent by members of the public who made contact via social media and the Hippoboscidae and Nycteribiidae Recording Scheme https://dipterists.org.uk/hippoboscidae-scheme/home, last accessed 12 February 2025) to request help with species identification. Additional flies were received from entomologists.

The hippoboscids were identified using a binocular microscope with reference to keys and species descriptions (Hill, 1964; Hutson, 1981, 1984; Maa, 1964, 1966, 1969a; Maa & Petersen, 1987; Oboňa et al., 2022). Three records of *Pseudolynchia canariensis* and one of *Ornithomya fringillina*, clearly identifiable from photographs, were included in the analyses despite the specimens being lost.

Data for historical comparisons were obtained from a previous study of the *Ornithomya* species in the UK and Ireland (Hill, 1962a), that used records that the author considered reliable together with his identification of flies in his own and other collections. These data, together with that from a range of other publications, including many that would be classed as ‘grey literature’, such as bird observatory and bird ringing group reports, were used to produce a comprehensive list of previously reported host interactions for the region. A list of all the references consulted can be found in the Supporting Information S1. Where possible, the original publications were reviewed and, if considered reliable, were given priority over more recent decisions. For example, Thompson refuted Theobald's claim that *Lipoptena cervi* was found on ‘fowls’ (domestic chickens), but the original report is accompanied by an illustration of an alate *L. cervi* (under the synonym *Ornithobia pallida* (Meigen)), and therefore the record is retained in the historical dataset used for comparisons (Theobald, 1896; Thompson, 1954). The taxonomy of all species was updated; for example, the misspelling of *Ornithomya* as ‘*Ornithomyia*’ was corrected (Maa, 1965); records of *Ornithomyia lagopodis* (Sharp) were updated to *Ornithomya chloropus* (Bergroth) (Hill, 1964). Corrections were made to records where necessary: for example, the only UK record of *Olfersia spinifera* (Leach) (Diptera: Hippoboscidae) was initially reported as being from a Magnificent Frigatebird *Fregata magnificens* Mathews (Suliformes: Fregatidae) (Graham et al., 1954), but later examination of the skin concluded that the host was an Ascension Frigatebird *Fregata aquila* (L.) (Suliformes: Fregatidae) (Chalmers, 2013).

The following groups of flies were excluded from the analyses of current host—parasite associations: flies originating from outside the study region or from before 2020, any flies that were free‐flying or when the host species was uncertain, and flies that were too damaged to be identified to species.

A total list of 282 potential host species was compiled to produce a standard matrix for the analyses (see Table S2). The potential avian host species were based on the British Trust for Ornithology's ‘Alphabetical list of regularly occurring UK bird species’ (https://www.bto.org/understanding-birds/birdfacts/about-birdfacts/alphabetical-list-regularly-occurring-uk-bird-species, last accessed 4 November 2024) with the addition of domestic chicken *Gallus gallus domesticus* L. (Galliformes: Phasianidae) (Theobald, 1896), and rare and vagrant bird species on which louse flies have been found in the region: Great Snipe *Gallinago media* (Latham) (Charadriiformes: Scolopacidae) (this study), Ascension Frigatebird (Chalmers, 2013; Graham et al., 1954), Little Bittern *Botaurus minutus* (L.) (Pelecaniformes: Ardeidae) (Thompson, 1955), Red‐footed Falcon *Falco vespertinus* L. (Falconiformes: Falconidae) (Hill, 1962a), Paddyfield Warbler *Acrocephalus agricola* (Jerdon) (Passeriformes: Acrocephalidae) (Wawman, 2024), White's Thrush *Zoothera aurea* (Holandre) (Passeriformes: Turdidae) (Hill, 1962a), and Citrine Wagtail *Motacilla citreola* Pallas (Passeriformes: Motacillidae) (Corbet, 1955). Redpolls (Passeriformes: Fringillidae), whether recorded as Common Redpoll *Acanthis flammea* (L.), Lesser Redpoll *Acanthis cabaret* (P.L. Statius Müller), *or as ‘redpoll’*, were merged to form a single species, Redpoll, *Acanthis flammea* for the analyses in line with current understanding of their taxonomy (Funk et al., 2021; Mason & Taylor, 2015) and earlier recording of this host in the publications related to hippoboscid host species in the region. Mammalian species were added only if hippoboscids have been collected from them in the region.

In the tables and plots, avian species are listed according to the British Ornithological Union's List (British Ornithologists' Union, 2024), which places the species in taxonomic order, apart from the merger of Redpolls. Mammal species were added to the end of the list and at the right‐hand side of the bipartite plots.

The data were analysed in R version 4.2.1 (R Development Core Team, 2022). The packages dplyr, version 1.1.4 (Wickham, Francois, et al., 2023), lubridate, version 1.9.3 (Grolemund & Wickham, 2011), and tidyr, version 1.3.0 (Wickham, Vaughan, & Girlich, 2023) were used to process the data prior to the analyses.

Bird host species weights (see Table S2) were obtained from the BTO's Bird Ringers' App (BTO IS and Demography Team, 2016). The relationship between the sympatric fly species (*O. fringillina* and *O. avicularia*) and their hosts' masses was analysed using a binomial generalized linear model (glm), fly species ~ host mass, with a clog‐log link function using the package arm version 1.13—1 (Gelman & Su, 2022). Other glms, namely, fly species ~ host mass + family and fly species ~ host mass * bird family, were used to explore the relationships between parasites and hosts.

The bipartite plots and network analyses were performed using the package bipartite version 2.18 (Dormann et al., 2022). As the main metrics of network structure are strongly affected by the size of the interaction matrix (Blüthgen et al., 2007; Morris et al., 2014), the comparison of the networks was limited to the three generalist *Ornithomya* species, with reliable data available from the earlier study (Hill, 1962a), and the same list of host species was used for both when plotting the bipartite diagrams. The number of interactions was equalized by waiting until the Mapping Project had exactly the same number of flies of the same three *Ornithomya* species as in the 1962 study. In all calculations of network metrics, the full web was used, by using the code ‘emptylist = FALSE’ during the production of the matrix, and ‘empty.web = FALSE’ in the calculations. However, as bipartite does not support the retention of empty rows and columns in the species level metrics, the default ‘empty.web’ = TRUE was used.

Producing bipartite plots to clearly show all of the interactions in the networks proved challenging because of the large dataset. Various methods were attempted, including plotting all species interactions on a 1:1 basis and/or reducing the number of host species by using family or order and grouping families together, but none proved satisfactory. The plots included in this publication are, therefore, a compromise in which, for the comparison of the current dataset with the data from the 1962 paper on the *Ornithomya* species (Hill, 1962a), in order to decrease the size of the plots, the host species are plotted by family with some related families, with similar ecologies grouped, and all of the Passeriformes placed in one group, as listed in Table S2. The group ‘waders’ contains the birds in the order Charadriiformes in the families Haematopodidae, Recurvirostridae, Charadriidae and Scolopacidae. The seabird group combines two families from the order Suliformes: the Sulidae (gannets) and Phalacrocoracidae (shags and cormorants), with one from the order Charadriiformes, the Alcidae (auks), one from the order Phaethontiformes, the Phaethontidae (divers), and two families from the order Procellariiformes: the Hydrobatidae (petrels) and the Procellariidae (shearwaters and fulmars). These related groups of species have similar ecology and would be expected to have similar chances of coming into contact with louse fly species.

## RESULTS

### Current data host—parasite associations

A total of 4365 flies and puparia of 12 species were received for identification, by the cut‐off date of 18 January 2025, of which 4085 could be linked to 117 host species (Table 1). Of the 212 host—parasite interactions, 70 were previously unreported in the region. A table of references showing all previously reliably reported host—Hippoboscidae associations, and those from this study, is shown in Table S3. The newly recorded interactions included the first record of the Pigeon Louse Fly *Pseudolynchia canariensis* in association with its host the feral pigeon *Columba livia domestica* J.F. Gmelin (Columbiformes: Columbidae), it having only previously been reported as free‐flying individuals in the region. Potentially important globally previously unreported interactions included (i) eight flies, of two *Ornithomya* species, found on seven individual gulls (Charadriiformes: Laridae), of three species, at six different sites, and (ii) alate Deer Keds *Lipoptena cervi* on three species of passerine.

**TABLE 1:** All Hippoboscidae‐host species associations recorded in the United Kingdom, Republic of Ireland and Isle of Man, from 2020 up until 18 January 2025, showing the total number of Hippoboscidae of each species taken from each host species.

Family	Binomial	Common name	Generalist louse flies				Stenoxenous louse flies					Rare	Keds			
			*Ornithomya avicularia*	*Ornithomya chloropus*	*Ornithomya fringillina*	*Ornithomya* sp.	*Ornithomya biloba*	*Crataerina pallida*	*Stenepteryx hirundinis*	*Pseudolynchia canariensis*	*Pseudolynchia garzettae*	*Icosta minor*	*Hippobosca equina*	*Lipotena cervi*	*Melophagus ovinus*	TOTAL
Phasianidae	*Lagopus lagopus*	Red Grouse		2												2
Phasianidae	*Alectoris rufa*	Red‐legged Partridge	1													1
Phasianidae	*Gallus gallus domesticus*	Chicken	1													1
Caprimulgidae	*Caprimulgus europaeus*	Nightjar	**2**								3					5
Apodidae	*Apus apus*	Swift						81	1							82
Cuculidae	*Cuculus canorus*	Cuckoo	2													2
Columbidae	*Columba livia*	Rock Dove			**1**											1
Columbidae	*Columba livia domestica*	Feral pigeon	1							**6**						7
Columbidae	*Columba oenas*	Stock Dove	**20**	**2**												22
Columbidae	*Columba palumbus*	Woodpigeon	**49**													49
Columbidae	*Streptopelia decaocto*	Collared Dove	3													3
Haematopodidae	*Haematopus ostralegus*	Oystercatcher		6	**1**											7
Charadriidae	*Vanellus vanellus*	Lapwing		30												30
Charadriidae	*Pluvialis apricaria*	Golden Plover		14												14
Charadriidae	*Charadrius hiaticula*	Ringed Plover		1												1
Scolopacidae	*Numenius arquata*	Curlew		11												11
Scolopacidae	*Limosa limosa*	Black‐tailed Godwit		**1**												1
Scolopacidae	*Arenaria interpres*	Turnstone		**2**												2
Scolopacidae	*Calidris alpina*	Dunlin		19												19
Scolopacidae	*Lymnocryptes minimus*	Jack Snipe		**2**												2
Scolopacidae	*Gallinago media*	Great Snipe		**2**												2
Scolopacidae	*Actitis hypoleucos*	Common Sandpiper	**1**	1												2
Scolopacidae	*Tringa totanus*	Redshank		1												1
Laridae	*Larus marinus*	Great Black‐backed Gull		**1**												1
Laridae	*Larus argentatus*	Herring Gull	**2**													2
Laridae	*Larus fuscus*	Lesser Black‐backed Gull	**5**													5
Laridae	*Sterna paradisaea*	Arctic Tern		**1**												1
Stercorariidae	*Stercorarius skua*	Great Skua		**1**												1
Pandionidae	*Pandion haliaetus*	Osprey	**2**													2
Accipitridae	*Aquila chrysaetos*	Golden Eagle		**1**												1
Accipitridae	*Accipiter nisus*	Sparrowhawk	24	1	**1**			**1**								27
Accipitridae	*Accipiter gentilis*	Goshawk	**9**													9
Accipitridae	*Circus cyaneus*	Hen Harrier		**24**												24
Accipitridae	*Milvus milvus*	Red Kite	**2**													2
Accipitridae	*Buteo buteo*	Buzzard	11													11
Tytonidae	*Tyto alba*	Barn Owl	75	**11**				**2**								88
Strigidae	*Athene noctua*	Little Owl	7													7
Strigidae	*Asio otus*	Long‐eared Owl	29	4												33
Strigidae	*Asio flammeus*	Short‐eared Owl	1	6												7
Strigidae	*Strix aluco*	Tawny Owl	22													22
Picidae	*Jynx torquilla*	Wryneck		**1**												1
Picidae	*Dendrocopos major*	Great Spotted Woodpecker	80						**1**							81
Picidae	*Picus viridis*	Green Woodpecker	21													21
Falconidae	*Falco tinnunculus*	Kestrel	12	9												21
Falconidae	*Falco columbarius*	Merlin	2	224												226
Falconidae	*Falco subbuteo*	Hobby	**5**					**1**								6
Falconidae	*Falco peregrinus*	Peregrine	2													2
Corvidae	*Garrulus glandarius*	Jay	5													5
Corvidae	*Pica pica*	Magpie	29													29
Corvidae	*Pyrrhocorax pyrrhocorax*	Chough	**26**													26
Corvidae	*Coloeus monedula*	Jackdaw	74													74
Corvidae	*Corvus frugilegus*	Rook	38													38
Corvidae	*Corvus corone*	Carrion Crow	51													51
Corvidae	*Corvus cornix*	Hooded Crow	**7**	**1**												8
Paridae	*Periparus ater*	Coal Tit			**6**									**1**		7
Paridae	*Poecile montanus*	Willow Tit			**1**											1
Paridae	*Cyanistes caeruleus*	Blue Tit	9	**2**	47											58
Paridae	*Parus major*	Great Tit	22	16	37											75
Panuridae	*Panurus biarmicus*	Bearded Tit				**1**										1
Alaudidae	*Alauda arvensis*	Skylark		4	1											5
Hirundinidae	*Riparia riparia*	Sand Martin		3			2		37							42
Hirundinidae	*Hirundo rustica*	Swallow	4	3	2		45		3							57
Hirundinidae	*Delichon urbicum*	House Martin						3	211							214
Cettiidae	*Cettia cetti*	Cetti's Warbler			**4**											4
Aegithalidae	*Aegithalos caudatus*	Long‐tailed Tit	1		6											7
Phylloscopidae	*Phylloscopus trochilus*	Willow Warbler	1	4	91											96
Phylloscopidae	*Phylloscopus collybita*	Chiffchaff			33											33
Acrocephalidae	*Acrocephalus schoenobaenus*	Sedge Warbler	**5**	1	5							**1**				12
Acrocephalidae	*Acrocephalus scirpaceus*	Reed Warbler	23		44											67
Locustellidae	*Locustella naevia*	Grasshopper Warbler	**1**	**9**												10
Sylviidae	*Sylvia atricapilla*	Blackcap	**7**	**1**	46											54
Sylviidae	*Sylvia borin*	Garden Warbler	**2**		2											4
Sylviidae	*Curruca curruca*	Lesser Whitethroat	1													1
Sylviidae	*Curruca communis*	Whitethroat	1	2	36											39
Sylviidae	*Curruca undata*	Dartford Warbler			1											1
Regulidae	*Regulus ignicapilla*	Firecrest			**2**											2
Regulidae	*Regulus regulus*	Goldcrest			49									**1**		50
Troglodytidae	*Troglodytes troglodytes*	Wren	1	3	10											14
Sittidae	*Sitta europaea*	Nuthatch	1		2											3
Certhiidae	*Certhia familiaris*	Treecreeper			10											10
Sturnidae	*Sturnus vulgaris*	Starling	146	21												167
Turdidae	*Turdus merula*	Blackbird	331	41	**7**	1										380
Turdidae	*Turdus iliacus*	Redwing	2	1										**1**		4
Turdidae	*Turdus philomelos*	Song Thrush	35	4	**4**											43
Muscicapidae	*Muscicapa striata*	Spotted Flycatcher	1	1	1											3
Muscicapidae	*Erithacus rubecula*	Robin	23	17	132											172
Muscicapidae	*Ficedula hypoleuca*	Pied Flycatcher	**1**	1												2
Muscicapidae	*Phoenicurus phoenicurus*	Redstart	**1**		**1**											2
Muscicapidae	*Saxicola rubetra*	Whinchat		3												3
Muscicapidae	*Saxicola rubicola*	Stonechat		**4**	2											6
Muscicapidae	*Oenanthe oenanthe*	Wheatear		39	1											40
Passeridae	*Passer domesticus*	House Sparrow	146	65	44											255
Passeridae	*Passer montanus*	Tree Sparrow	3		4											7
Prunellidae	*Prunella modularis*	Dunnock	70	9	73											152
Motacillidae	*Motacilla flava*	Yellow Wagtail		1												1
Motacillidae	*Motacilla cinerea*	Grey Wagtail	1	2												3
Motacillidae	*Motacilla alba*	Pied Wagtail	4	20	1											25
Motacillidae	*Anthus pratensis*	Meadow Pipit	**3**	151	4											158
Motacillidae	*Anthus trivialis*	Tree Pipit	**2**	11	2											15
Motacillidae	*Anthus petrosus*	Rock Pipit		5	1											6
Fringillidae	*Fringilla coelebs*	Chaffinch	69	**45**	70											184
Fringillidae	*Pyrrhula pyrrhula*	Bullfinch	8	1	**5**											14
Fringillidae	*Chloris chloris*	Greenfinch	36	**2**	20											58
Fringillidae	*Linaria cannabina*	Linnet	6	5	5											16
Fringillidae	*Acanthis flammea*	Redpoll		2	**5**											7
Fringillidae	*Loxia curvirostra*	Crossbill	**1**													1
Fringillidae	*Carduelis carduelis*	Goldfinch	**19**	**35**	62											116
Fringillidae	*Spinus spinus*	Siskin	**13**	**60**	**31**											104
Calcariidae	*Emberiza calandra*	Corn Bunting	2													2
Calcariidae	*Emberiza citrinella*	Yellowhammer	**4**		7											11
Calcariidae	*Emberiza schoeniclus*	Reed Bunting	**8**		3											11
Cervidae	*Dama dama*	Fallow Deer												13		13
Cervidae	*Capreolus capreolus*	Roe Deer												36		36
Cervidae	*Cervus elaphus*	Red Deer												15		15
Bovidae	*Ovis aries*	Sheep													55	55
Equidae	*Equus ferus caballus*	Horse											4	**2**		6
																
Hominidae	*Homo sapiens*	Human	1	**1**										20		22
	Total number of Hippoboscidae		1636	974	923	2	47	88	253	6	3	1	4	89	55	4081
	Total number of interactions		74	64	47	2	2	5	5	1	1	1	1	8	1	212
	**New host species interactions**		**24**	**22**	**13**	1*	**0**	**3**	**1**	**1**	**0**	**1**	**0**	**4**	**0**	69*
	Previously published associations		68	71	45		3	4	4	0	1	2	4	9	1	219
	Total known host associations		92	93	58		3	7	5	1	1	3	4	13	1	288*

*Note*: The three generalist *Ornithomya* species totals are shown in the left‐hand columns, the keds in the right‐hand columns, with the most host specific species found on birds in the centre. New host—parasite interactions are highlighted in red bold typeface. Rock Dove *Columbia livia* and feral pigeon *Columbia livia domestica* are listed separately because of their differing habitats, although they are only counted as a single species. Redpoll *Acanthis flammea* is treated as a single species. The underlined totals of previously published associations (sources listed in Supplementary information S1) and overall total also include the following seven interactions that are not included within the table, as the louse fly species were not caught during the study: *Icosta ardae* with Bittern *Botaurus stellaris*, Little Bittern *Botaurus minutus*, and Purple Heron *Ardea purpurea; Olfersia spinifera* with Ascension Frigatebird *Fregata aquila; Ornithophila metallica* with Whitethroat *Curruca communis*; the two accidental imports of *Ornithophila gestroi* with Red‐billed Leiothrix *Leiothrix lutea* and Ring‐necked Parakeet *Psittiacula krameri*. One damaged fly and one puparium were not identifiable to species level are included as *Ornithomya* sp. and these are excluded from the totals indicated by an asterisk.

Bipartite plots showed a complex web of interactions between species, including *Homo sapiens*, who were reported as being bitten by *Lipoptena cervi*.

Louse flies were recorded from all of the most common families of passerines, with the exception of the Cinclidae, of which the only species in the region is its only aquatic songbird, the Dipper (White‐throated Dipper) *Cinclus cinclus* (L.) (Passeriformes: Cinclidae).

The other two passerine families with no flies recorded were the Bombycillidae represented by the irruptive winter migrant, Waxwing (Bohemian Waxwing) *Bombycilla garrulus* (L.) (Passeriformes: Bombycillidae), which is almost always caught outside the louse fly season, and Oriolidae, a family containing only the Golden Oriole *Oriolus oriolus* (L.) (Passeriformes: Oriolidae), which no longer breeds in the region. A puparium of one of the *Ornithomya* spp. was received from a Bearded Tit *Panurus biarmicus* (L.) (Passeriformes: Panuridae), but a fly failed to emerge and could not be identified to species, either morphologically or with DNA sequencing.

Amongst the other avian families, louse flies were absent from the aquatic families, Anatidae (Anseriformes) (geese, swans and ducks), Rallidae (Gruiformes) (rails), Podicipedidae (Podicipediformes) (grebes), Alcidae (auks), Phaethontidae (divers/loons), Hydrobatidae (petrels), Procellariidae (shearwaters), Sulidae (gannets), Phalacrocoracidae (cormorants), and Ardeidae (Pelecaniformes) (herons, bitterns, and egrets). This was despite ringers being encouraged to look for first records of louse flies from these hosts and large numbers being ringed by volunteers, with the exception of the Phaethontidae and Podicipedidae, which are ringed in very low numbers in the region.

After removal of flies that were free flying, or where the host was uncertain (*n* = 276), a puparium from a Bearded Tit and a damaged fly that could only be identified to genus, 4079 Hippoboscidae of 12 species, from 116 host species, was included in the analysis of current host—parasite distributions.

Selected network metrics for all the species of Hippoboscidae collected for this study are shown in Table 2, with the full results in Table S4. The degree is the number of host associations for the ectoparasite species, and the species specificity index (SSI) is a coefficient of variation of interactions, normalized between 0 and 1, which does not take into account the species relationships such as taxonomy or ecology. A high SSI indicates a high level of specificity but is unreliable if only one interaction is present in the matrix, as seen here in the case of *Icosta minor* (Bigot) (Diptera: Hippoboscidae). The results show that *O. avicularia*, *O. chloropus* and *O. fringillina* are generalists and suggest that *L. cervi* may also be less host specific than the remaining species; however, as they are based on occurrence only, these metrics give no indication of the level of fitness of a species on a given host species or whether it is even able to survive on it. The figures suggest that the other species are more or less host specific. The host species totals of the species with a high SSI that is less than one are useful in suggesting possible mechanisms for the presence of other ectoparasite species. For example, *C. pallida* was found on Hobby *Falco subbuteo* L. (Falconiformes: Falconidae) and Sparrowhawk *Accipiter nisus* (L.) (Accipitriformes: Accipitridae), both of which are bird predators, and it is likely that the flies transferred to them from their prey.

**TABLE 2:** Selected network metrics for all the species of Hippoboscidae collected for this study, of Hippoboscidae in the United Kingdom, Republic of Ireland, and Isle of Man, from 2020 to 2024, calculated in the R package bipartite (Dormann et al., 2022).

Hippoboscid species	Degree	Species specificity index
*Ornithomya avicularia*	74	0.255
*Ornithomya chloropus*	64	0.298
*Ornithomya fringillina*	47	0.240
*Ornithomya biloba*	2	0.958
*Crataerina pallida*	5	0.921
*Stenepteryx hirundinis*	5	0.845
*Pseudolynchia canariensis*	1	1
*Pseudolynchia garzettae*	1	1
*Icosta minor*	1	1
*Hippobosca equina*	1	1
*Lipoptena cervi*	8	0.508
*Melophagus ovinus*	1	1

*Note*: The degree is the number of host associations for the ectoparasite species, and the species specificity index (SSI) is a coefficient of variation of interactions, normalized between 0 and 1, which does not take the species relationships such as taxonomy or ecology into account. A high SSI indicates a high level of specificity but is unreliable if only one interaction is present in the matrix, as seen here in the case of *Icosta minor*. The results show that *Ornithomya avicularia*, *O. chloropus*, and *O. fringillina* are generalists and suggest that *Lipoptena cervi* may also be less host specific than the remaining species but given no indication of whether hippoboscids are able to survive on a given host. The full network metrics are available in Table S4.

**FIGURE 1:**
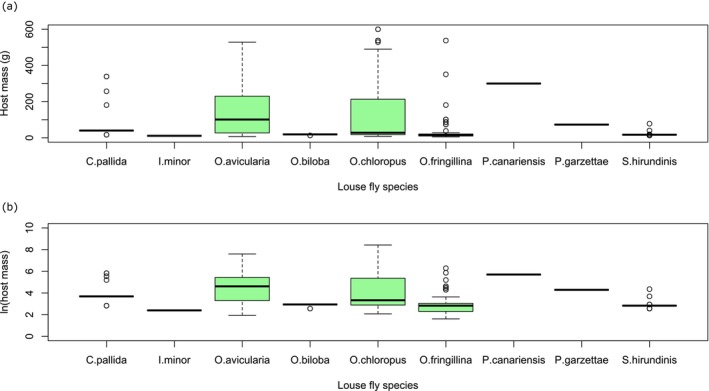
Boxplots showing the relationship between mean host mass and louse fly species in this study of Hippoboscidae from the United Kingdom, Republic of Ireland and Isle of Man from 2020 to 2024: (a) with host mass capped at 600 g; (b) with host mass expressed as a natural logarithm. Both show that only the three generalist species, *Ornithomya avicularia, Ornithomya chloropus* and *Ornithomya fringillina* are frequently found on a range species of different masses, with *O. fringillina* parasitizing smaller host species. The other species (*Crataerina pallida, Icosta minor, Ornithomya biloba, Pseudolynchia canariensis, Pseudolynchia garzettae* and *Stenepteryx hirundinis*) are monoxenous and stenoxenous.

**FIGURE 2:**
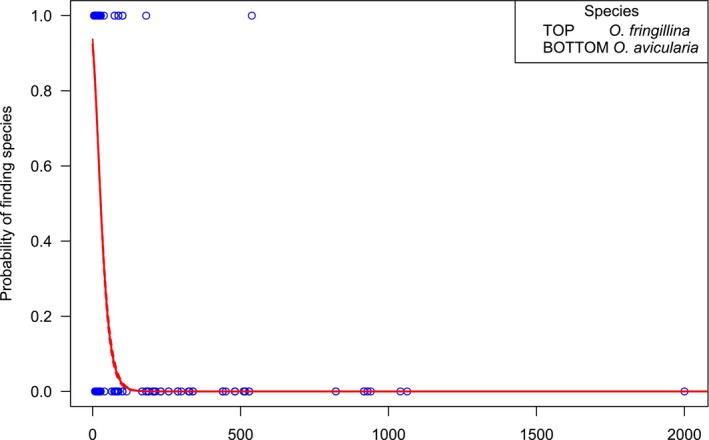
Hippoboscidae from the United Kingdom, Republic of Ireland and Isle of Man from 2020 to 2024: Probability of finding the sympatric species *Ornithomya fringillina* (top) or *Ornithomya avicularia* (bottom) by bird host mass in grams (solid red line), with upper and lower standard error lines (dotted lines) predicted from a binomial glm: Glm (formula = fly species ~ bird mass, family = binomial) (link = cloglog) (*p* < 2e^−16^).

For most species of Hippoboscidae, the host masses fell within a narrow range, with this being especially narrow for most stenoxenous species but wider for the generalist species (Figure 1). A glm showed that the niches of the two sympatric generalist species, *O. avicularia* and *O. fringillina*, were largely separated by the weight of their hosts (Figure 2), with a low probability (*p* < 0.05) of finding *O. fringillina* on birds with a mass of over 80 g and a very low probability (*p* < 0.01) of finding it on birds with a mass of 100 g or more (model prediction probability *p* < 0.001).

No bird families were significant (all *p* > 0.990), whether included as an interaction term with mass or in a separate model against the species. However, the residuals were overdispersed, and the model fit was poor (null deviance = 3209.8 on 2462 degrees of freedom, residual deviance = 1455.1 on 2410 degrees of freedom), and there was a high degree of correlation between bird family and host mass (Kruskal‐Wallis chi‐squared = 201.28, degrees of freedom = 34, *p*‐value <2.2e‐16).

### Comparison with data from Hill, 1962

Totally, 3533 flies in the genus *Ornithomya* (*O. avicularia n* = 1636, *O. chloropus n* = 974, and *O. fringillina n* = 923) were used to compare the data with the same number of flies in the earlier dataset (*O. avicularia n* = 1030, *O. chloropus n* = 2083 after correcting the total, and *O. fringillina n* = 420) (Hill, 1962a).

A comparison of the two sets of data, current and pre‐1962, (Hill, 1962a) for the *Ornithomya* species (Table 3) showed that the total number of interactions for all three generalist species had increased during the 60 years since the previous study, with the connectance, or proportion of possible interactions realized, increasing from 0.147 to 0.219 of possible interactions detected. All three species increased their number of interactions (network degree): *O. avicularia* from 43 to 74, *O. chloropus* from 56 to 64, and *O. fringillina* from 25 to 47. The SSI for all three species decreased, indicating a decrease in host specificity. A full table of all the network metrics calculated by the bipartite package for both networks of species can be found in Table S5.

**TABLE 3:** Selected network metrics comparing the Hippoboscidae‐host interactions of hippoboscids collected for this study, of Hippoboscidae in the United Kingdom, Republic of Ireland, and Isle of Man, from 2020 to 2024, to those from the 1962 study (Hill, 1962a) for the three generalist species in the genus *Ornithomya*.

	Hill, 1962			2020—2024		
	n	Degree	SSI	n	Degree	SSI
Species						
*Ornithomya avicularia*	1030	43	0.438	1636	74	0.254
*Ornithomya chloropus*	2083	56	0.416	974	64	0.297
*Ornithomya fringillina*	420	25	0.620	923	47	0.239
Total	3533			3533		
Network metrics						
Total interactions	124			185		
Connectance	0.147			0.219		

*Note*: The values show an increase in the number of host species and the connectance (the number of potential interactions that are realised), together with a decrease in the species specificity index (SSI) of all three species. The full network metrics are available in Table S5.

**FIGURE 3:**
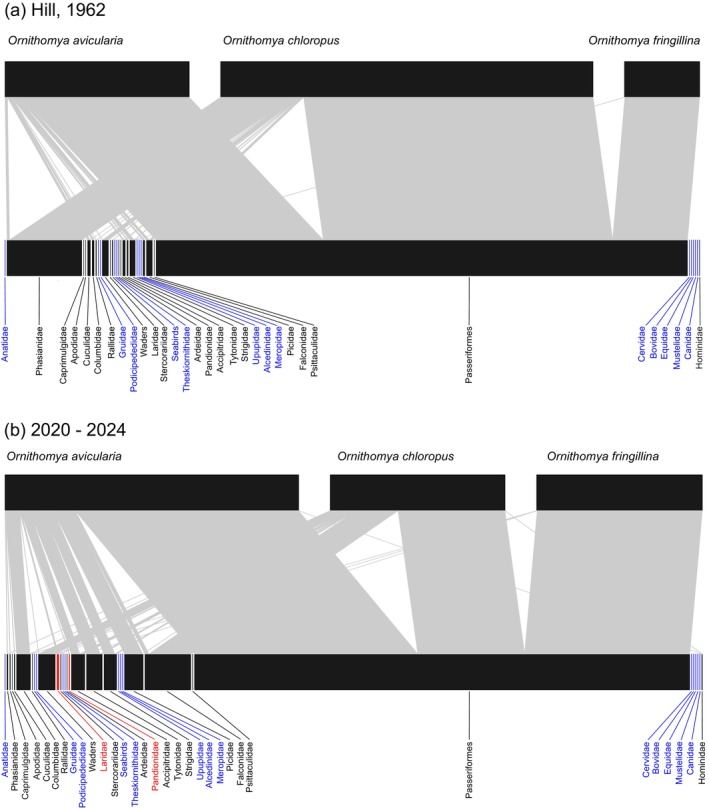
Bipartite plots showing the host parasite relations of the three most common *Ornithomya* species, plotted against hosts. The ectoparasites are shown at the top with the hosts along the bottom. (a) records from Hill (1962), (b) the current study of Hippoboscidae from the United Kingdom, Republic of Ireland and Isle of Man from 2020 to 2024. In order to decrease the size of the plots, the host species are plotted by family with some related families, with similar ecologies grouped, and all of the Passeriformes placed in one group, as listed in Table S2. The group ‘waders’ contains the birds in the order Charadriiformes in the families Haematopodidae, Recurvirostridae, Charadriidae and Scolopacide. The seabird group combines two families from the order Suliformes the Sulidae (gannets) and Phalacrocoracidae (shags and cormorants), with one from the order Charadriiformes, the Alcidae (auks), one from the order Phaethontiformes the Phaethontidae (divers), and two families from the order Procellariiformes the Hydrobatidae (petrels) and the Procellariidae (shearwaters and fulmars). These related groups of species have similar ecology and would be expected to have similar chances of coming into contact with louse fly species. The two avian families highlighted in red gulls (Charadriiformes: Laridae) and Osprey (Accipitriformes: Pandionidae) in Figure 3b are those with first records of louse flies for the UK in this study. The significance of the flies on Osprey is uncertain as the host was a sick juvenile taken to a rescue centre. Seabirds, and the bird families Anatidae (Anseriformes), Gruidae (Gruiformes), Podicipedidae (Podicipediformes), Threskiornithidae (Pelecaniformes), Upupidae (Bucerotiformes), Alcedinidae (Coraciiformes), and Meropidae (Coraciiformes), that have never been recorded as having louse flies in the region are labelled in blue. Note the aquatic nature of most of the avian families (Anatidae, Gruidae, Podicipedidae, seabirds, Threskiornithidae and Alcedinidae) that do not have louse flies. Note also that although they are highlighted in blue, to indicate an absence of louse flies, all of the mammalian families have been recorded as hosts of keds, the group of Hippoboscidae that parasitize mammals. Families labelled in black are those with published host associations of any species of louse fly in the region. This includes records of these three species of *Ornithomya* from other studies, as well as those of other species of louse fly. Bird hosts' ‘families’ are in order listed according to the British Ornithological Union's List (British Ornithologists' Union, 2024) which places the species in taxonomic order and the relevant mammalian host families have been added on the right hand side.

Bipartite plots made from both sets of data, for the *Ornithomya* species, show a complex web of interactions even when plotted at the level of grouped families (Figure 3). The 1960s web is dominated by the presence of *O. chloropus*, whereas in the 2020—2024 web, most of the interactions are with *O. avicularia*. The large number of interactions with birds in the family Phasianidae in the 1960s data suggests that a lot of Hill's specimens came from gamebirds that had been shot. Notable recent additions to the list of species interactions include those between gulls (Laridae) and *O. avicularia* and *O. chloropus*.

## DISCUSSION

A total of 4365 flies and puparia of 12 species were received from 117 host species (Table 1). Of these host—parasite interactions, 70 were previously unreported in the region, including the first globally reported detections of louse flies on three species of gulls and of the Deer Ked *L. cervi* on three species of birds (Table S3). Comparisons with data from the 1962 paper (Hill, 1962a) showed that all three generalist species in the genus *Ornithomya* had increased the number of host species with which they were associated. However, even for the genus *Ornithomya*, these analyses underestimate the true increase in complexity as they do not include the new colonist, *O. biloba*, or take into account interactions of their hosts with other hippoboscids.

Whereas the monoxenous and stenoxenous species are separated by their host species, the niche separation of the generalist species is more complex. A mark‐recapture study of *O. chloropus* on Fair Isle (originally identified as *O. fringillina* before the species were revised in 1962 (Hill, 1962b)) concluded that flies that were released without a host attached themselves to the first bird that they could regardless of species and did not attempt to return to the original host species, although flies did move between hosts (Corbet, 1956). *Ornithomya chloropus* is largely separated from the other two species by its preference for higher latitudes and northern altitudes, but *O. avicularia* and *O. fringillina* are sympatric across most of their range (Wawman, 2025a). However, the smaller species, *O. fringillina*, has a preference for smaller birds and *O. avicularia* for larger bird species. This association between the smaller louse fly, *O. fringillina*, and smaller hosts, and of *O. avicularia* with larger hosts has been reported previously (Hill, 1962a; Lehikoinen et al., 2021).

In this region, the Laridae (gulls) are the only aquatic species which have louse flies. This study found eight louse flies on gulls: five *O. avicularia* on Lesser Black‐backed Gull *Larus fuscus* L. (Charadriiformes: Laridae), two on Herring Gull *L. argentatus* Pontoppidan (Charadriiformes: Laridae), and one *O. chloropus* on Great Black‐backed Gull *L. marinus* L. (Charadriiformes: Laridae). Worldwide, there are only two previous records of louse flies on gulls. The first was of ‘*Ornithomya fringillina*’ on a gull *Larus* sp. in Denmark (Johnsen, 1948); but, unfortunately, only two species of *Ornithomya* were reported, and it is probable that Johnsen did not recognize *O. chloropus*, as the two species were not often separated until the genus *Ornithomya* was revised in 1962 (Hill, 1962b). The second record is of *O. avicularia* on a Black‐headed Gull *Chroicocephalus ridibundus* (L.) (Charadriiformes: Laridae) in the Netherlands (Van Eck & Van Den Broek, 1969).

Louse flies should be obvious on the pale plumage of gulls, and the total number of gulls of these three species ringed annually in the UK is now only around a third of what it was in the 1960s (Robinson et al., 2024), so it is unlikely that louse flies would have gone unrecorded if they were present in the 1950s and early 1960s. Gulls have only colonized urban areas in the last 60 years (Rock & Vaughan, 2013), and this, with the associated change in diet to anthropogenic sources, such as food waste from landfill sites (O'Hanlon et al., 2025), has led to gulls mixing with other avian species. Carrion Crows *Corvus corone* L. (Passeriformes: Corvidae) and Starlings *Sturnus vulgaris* L. (Passeriformes: Sturnidae) are frequently infested with louse flies that could move to new hosts such as gulls.

With the change to anthropogenic food sources, gulls spend less time fishing. Some of the species of louse fly, such as those in the genus *Olfersia* (Diptera: Hippoboscidae), for example, *Olf. spinifera*, found on frigatebirds *Fregata* spp. Lacépède (Suliformes: Fregatidae), pelicans *Pelecanus* spp. L. (Pelecaniformes: Pelecanidae), cormorants (Phalacrocoracidae), and boobies Sula spp. Brisson (Suliformes: Sulidae), and *Olfersia fumipennis* (Sahlberg) (Diptera: Hippoboscidae), found on Osprey *Pandion haliaetus* (L.) (Accipitriformes: Pandionidae)—must be able to tolerate submersion, but it is possible that the UK louse flies cannot, as they were not found on truly aquatic host species and would not have been able to survive on gulls that regularly dived for fish.

In addition to various mammalian hosts, the Deer Ked, *L. cervi*, was found on the Passeriformes Goldcrest *Regulus regulus* (L.) (Regulidae), Coal Tit *Periparus ater* (L.) (Paridae), and Redwing Turdus iliacus L. (Turdidae). It has previously been recorded on other birds including Peregrine *Falco peregrinus* Tunstall (Falconiformes: Falconidae) in Denmark (Johnsen, 1948), Oriental Turtle Dove *Streptopelia orientalis* (Latham) (Columbiformes: Columbidae) in eastern Russia (Nartshuk et al., 2022), and a number of Passeriformes: Song Thrush *Turdus philomelos* C. L. Brehm (Turdidae) in Slovakia (Krišovský et al., 2024), and Rufous‐tailed Robin *Larvivora sibilans* Swinhoe (Muscicapidae), Marsh Tit *Poecile palustris* (L.) (Paridae), and Radde's Warbler *Phylloscopus schwarzi* (Radde) (Phylloscopidae) in Eastern Russia (Nartshuk et al., 2022). The presence of a parasite on a host does not prove that it is able to successfully parasitize it. All three of the specimens caught were still alate—*L. cervi* shed their wings upon finding a suitable host—which could be consistent with incidental findings, including being initially attracted to the bird ringers, rather than true examples of parasitism. However, the low number of reported interactions between *L. cervi* and birds may be a result of the historical tendency to study louse flies and keds separately, or because bird ringers collecting louse flies may not realize the significance of delate Deer Keds.

Species such as *L. cervi*, which is known to bite humans, and parasites present on urban host species such as feral pigeons and gulls, bring with them an increased risk of spreading diseases to humans, particularly in the presence of other vectors that bite humans, and they may act together to maintain enzootic cycles of disease, including potential zoonoses.

Clearly, there can be issues with comparing current data to that published 60 years ago. Some of the species from which ectoparasites were obtained recently have undergone marked population changes since the 1960s. For example, Red Kites *Milvus milvus* (L.) (Accipitriformes: Accipitridae) increased their breeding distribution — measured as their presence in 10 km squares—by 2035%, and Goshawk *Accipiter gentilis* (L.) (Accipitriformes: Accipitridae) by 1446%, whereas other species' numbers and ranges have declined (Balmer et al., 2013). Many of Hill's specimens were from museums and of unstated age, but this should not be a problem as the significant increase in regional temperatures only occurred after this period (Hartmann et al., 2013), as did gull's colonization of urban areas (Rock & Vaughan, 2013).

Amongst Hill's totals, there were flies from bird observatories and other bird ringers, but some of the host specimens were almost certainly from birds that had been shot, including corvids, Red Grouse *Lagopus lagopus scotica* (Latham) (Galliformes: Phasianidae), and other game birds. In the 2020—2024 study, the records are more widely distributed across the region, and very few were from hosts that had been shot. Bird ringers' capture methods have changed over the period, with mist nets, introduced in the late 1950s, replacing various traps as the main capture method for most ringers. Until at least the mid‐1970s, bird ringers in the region could use chloroform in a Fair Isle Apparatus (Williamson, 1954) or insecticidal powders to remove ectoparasites from wild birds (Spencer, 1976). All of these changes could easily cause a change in the proportions of host species and ectoparasites caught.

Some species of louse fly that might be expected in the region were not detected, although they may have been missed as only small numbers of their hosts are ringed. These species may have ‘missed the boat’— not arrived with the hosts that colonized the region— or ‘drowned on arrival’—arrived but failed to establish viable populations (MacLeod et al., 2010).

*Olfersia fumipennis* is commonly found on Osprey around the world, including other parts of Europe (Beuk, 2010; Hutson, 1984; Lehikoinen et al., 2021; Maa, 1969a; Reeves & Lloyd, 2019). Only *O. avicularia* was found parasitizing Osprey in this study, and the two flies were on a diseased juvenile taken to a rescue centre, and therefore of uncertain significance. Although there are currently around 300 pairs of Osprey breeding in the region, they became extinct in the UK in 1847, and slowly recolonized after a pair arrived naturally in Scotland in 1954. It is likely either no *Olf. fumipennis* were present, or that their population was too small for the parasites to establish. Osprey were also reintroduced into England in 2001 (https://www.roydennis.org/animals/raptors/osprey/species-recovery/scotland, last accessed 9th November 2024), and although these introduced Osprey were not treated for ectoparasites, none were observed (Roy Dennis, pers. comm.).

Similarly, *Icosta ardeae* (Macquart) (Diptera Hippoboscidae) is found on herons, bitterns and egrets (Pelecaniformes: Ardeidae) over large parts of the Palearctic, Nearctic, and Africa (Maa, 1969b) but has only previously been recorded in the region as a vagrant on three occasions (Chandler, 2024; Hutson, 1984). The number of potential host species and the size of their populations breeding in the region have increased markedly since the 1960s, especially for Little Egret *Egretta garzetta* (L.) and Bittern *Botaurus stellaris* (L.), with more recent colonisations by Great White Egret *Ardea alba* L. and Cattle Egret *Bubulcus ibis* L., but no *I. ardeae* were found in this study. This may be in part because most of these species are ringed as nestlings before they are fully feathered.

The stochastic nature of the records used in this study means that it is impossible to determine reliable infestation rates for any given species. The number of flies caught is likely to be influenced not only by the random sampling of birds and factors which cause flies to choose to leave their avian hosts, but by the visual acuity, agility, and determination of the bird ringers involved, whether they are ringing indoors where it is easier to trap flies which leave birds, or outside, and the time pressure under which they find themselves. Targeted sampling, using a knockdown method to trap louse flies in a controlled environment, would produce data that could be used more accurately to compare infestation rates.

Future research could include additional analyses of how the host ecology affects the likelihood of infestation by louse flies; for example, ticks have been found to be more common on ground‐feeding birds (Burnus et al., 2025). Continued monitoring to look for future host switches and range shifts may be important, especially as some of these species are known to vector diseases, and unless urgent action is taken to limit anthropogenic changes to the environment, further changes in ranges and host species are likely to occur. Continued specimen collection, followed by the use of molecular methods such as a metagenomics approach or targeted PCR to search for pathogenic microorganisms, and researching these species' vectorial capacity will be important in determining the risk of possible disease transmission both within and between species.

## AUTHOR CONTRIBUTIONS

**Denise C. Wawman:** Conceptualization; investigation; methodology; validation; data curation; formal analysis; visualization; project administration; resources; writing – original draft; writing – review and editing. **Adrian L. Smith:** Supervision; writing – review and editing. **Ben C. Sheldon:** Supervision; writing – review and editing.

## CONFLICT OF INTEREST STATEMENT

The authors declare no conflicts of interest.

## ETHICS STATEMENT

All wild birds were handled by bird ringers licensed by the British Trust for Ornithology.

## Supporting information


**Data S1**. Sources of information checked for host species records.

**Data S2**. Table of host species and their masses used in the models.

**Data S3**. Table of all known host‐Hippoboscidae interactions from the United Kingdom, Republic of Ireland and Isle of Man.

**Data S4**. A table of the network metrics calculated by the bipartite package for all species of Hippoboscidae in the current study: (a) species metrics, (b) network metrics.

**Data S5**. A table comparing the network metrics calculated by the bipartite package the three generalist species in the genus *Ornithomya* (*O. avicularia, O. chloropus* and *O. fringillina*) from the 1960s study (Hill, 1962a) and the current study: (a) species metrics, (b) network metrics.

## Data Availability

The data are available on the Dryad website (Wawman, 2025b) https://doi.org/10.5061/dryad.4b8gthtrj.
