# Supplementary material for: Citizen science reveals host‐switching in louse flies and keds (Diptera: Hippoboscidae) during a period of anthropogenic change
Source: Med Vet Entomol. 2025 Nov 1;40(2):305–22. doi: 10.1111/mve.70029 (PMC13140014; doi:10.1111/mve.70029)
Supplement: Supplementary file 1 — Data S1. Sources of information checked for host species records. [file MVE-40-305-s003.docx]

**S1. Sources of information checked for host species records**

**Publications**

Early systematic work by entomologists and museum curators, who examined large numbers of louse flies, which had been collected over previous decades (Thompson, 1953, 1954b, 1954a, 1955b, 1955c, 1955a; Bequaert, 1954; Hill, 1962; Hutson, 1984; O’Connor and Sleeman, 1987).

Small more intense studies of single species of flies on suitable hosts, for example, *Crataerina (Stenepteryx) hirundinis* on Hirundines (Summers, 1975) and Crataerina pallida on Swift *Apus apus* (Hutson, 1981).

Reports of new species in the United Kingdom and Republic of Ireland (Graham *et al.*, 1954; Lloyd-Evans, 1967; Thompson, 1968; Palmer, 1987; Harrow, 2021; Wawman, 2024).

Other peer reviewed literature: (Curtis, 1836; SMART, 1945; Kettle and Utsi, 1955; G. B. Corbet, 1956; Gordon B. Corbet, 1956; Hill, Wilson and Corbet, 1967; Smiddy, 1997; Denton, 2004; Smiddy and Sleeman, 2004; Turner and Mann, 2004; Harris, 2009; Gibson, Pilkington and Pemberton, 2010; Macdonald, 2022)

Books: (Theobald, 1896; Smart, 1939)

Letters: (White, 1789a, 1789b, 1789c)

Other published reports (grey literature, that is, literature which has not been through peer review). Only those with reports of Hippoboscidae are included:

Bird observatory reports (Williamson, 1949, 1950, 1952, 1954; Butterfield, 1951; Edwards, 1951, 1952; Thompson, 1952, 1964; Stansfield, 1954; Corbet, 1955; Ennion, 1955; G. Stansfield, 1955; Geoffrey Stansfield, 1955; Thompson and Beaumont, 1968; Waine, 1993, 1997, 1999, 2000, 2001, 2002, 2004, 2005, 2007, 2008, 2009, 2013, 2014).

Other bird reports, often from ringing groups, (Lloyd-Evans, 1967; Burn, 1968, 1969, 1970, 1971, 1972, 1973, 1974; Denton, 1977, 1978, 1979, 1980, 1974, 1975, 1976)

Reports of Diptera (Waterson, 1910; Ash, 1952, 1955; Ash and Monk, 1959; Beaumont, 1965; Cutts, 1971; Lane, 1978; Sellers and Redgate, 1992; Emley, 1992; Hancock, 1996, 1995; Chandler, 2009, 2021, 2023; Johnson, 2020).

**References**

Ash, J. (1952) ‘Records of Hippoboscidae (Dipt.) from Berkshire and Co. Durham in 1950, with notes on their bionomics.’, *The Entomologist’s Monthly Magazine*, lxxxviii, pp. 25–30.

Ash, J.S. (1955) ‘Some records of bird and mammal ectoparasites’, *The Entomologist’s Monthly Magazine*, xc1, pp. 64–65.

Ash, J.S. and Monk, J.F. (1959) ‘A collection of Ornithomyia spp. (Dipt., Hippoboscidae) from Oxfordshire’, *The Entomologist’s Monthly Magazine*, xcv(10th September), pp. 80–81.

Beaumont, H.E. (1965) ‘A review of the records of Yorkshire Hippoboscidae (Diptera)’, *Naturalist*, pp. 99–101.

Bequaert, J.C. (1954) ‘The Hippoboscidae or louse-flies (Diptera) of mammals and birds Part II. Taxonomy, evolution and revision of American genera and species’, *Entomologica Americana*, XXXIV, pp. 3-181+.

Burn, D.M. (1968) ‘Hippoboscidae - the dipterous flies parasitic on birds’, *Knaresborough Bird Ringing Station Report*, pp. 19–21.

Burn, D.M. (1969) ‘Hippoboscidae - the dipterous flies parasitic on birds’, *Knaresborough Bird Ringing Station Report*, pp. 12–13.

Burn, D.M. (1970) ‘Hippoboscidae - the dipterous flies parasitic on birds’, *Knaresborough Bird Ringing Station Report*, pp. 15–21.

Burn, D.M. (1971) ‘Hippoboscidae - the dipterous flies parasitic on birds’, *Knaresborough Bird Ringing Station Report*, pp. 35–39.

Burn, D.M. (1972) ‘Hippoboscidae - the dipterous flies parasitic on birds’, *Knaresborough Bird Ringing Station Report*, pp. 51–55.

Burn, D.M. (1973) ‘Hippoboscidae - the dipterous flies parasitic on birds’, *Knaresborough Bird Ringing Station Report*, pp. 35–42.

Burn, D.M. (1974) ‘Hippoboscidae - the dipterous parasitic flies parasitic on birds’, *Knaresborough Bird Ringing Station Report*, pp. 33–37.

Butterfield, A. (1951) ‘A Quantitative Analysis of the Results of the Ectoparasite Study.’, *Fair Isle Bird Observatory Annual Report 1951*, (37–38), pp. 38–41.

Chandler, P.J. (2009) ‘Ethel Katharine Pearce (1856-1940) and her contribution to dipterology’, *Dipterists Digest Second Series*, 16(2), pp. 117–146.

Chandler, P.J. (2021) ‘Hippoboscidae in Diptera species recorded at Windsor Forest and Great Park’, *Dipterists Digest*, 28(Supplement Appendix), p. 213.

Chandler, P.J. (2023) ‘An Update of the 1998 Checklist of Diptera of the British Isles [ updated 20 March 2023 ]’, pp. 1–440. Available at: https://dipterists.org.uk/sites/default/files/pdf/BRITISH ISLES CHECKLIST 2021_07.pdf.

Corbet, G. B. (1956) ‘The Life-History and Host-Relations of a Hippoboscid Fly Ornithomyia fringillina Curtis’, *The Journal of Animal Ecology*, 25(2), p. 403. Available at: https://doi.org/10.2307/1934.

Corbet, Gordon B. (1956) ‘The Phoresy of Mallophaga on a Population of Ornithomya fringillina Curtis (Dipt., Hippoboscidae)’, *The Entomologist’s Monthly Magazine*, xcii(September), pp. 207–211.

Corbet, G.C. (1955) ‘Research on Flat-flies at Fair Isle in 1954’, *Fair Isle Bird Observatory Bulletin*, 2(7), pp. 313–317. Available at: http://www.fairislebirdobs.co.uk/annual_reports.html.

Curtis, J. (1836) ‘Ornithomyia fringillina’, *British Entomology*, 13, pp. 582–3.

Cutts, D.B. (1971) ‘Some recent records of Hippoboscidae (Diptera), including Stenepteryx hirundinis L. from Swallow in V.C. 61’, *Bulletin of Hull Natural History Society*, pp. 3–7.

Denton, J. (2004) ‘The Forest Fly Hippobosca equina Linnaeua, 1958 (Diptera, Hippoboscidae) in North Hampshire’, *Dipterists Digest*, 11(1), p. 24. Available at: https://www.dipterists.org.uk/digest.

Denton, M.L. (1974) ‘Ectoparasites’, *Annual Report 1974 Huddersfield Birdwatchers’ Club*, pp. 9–12.

Denton, M.L. (1975) ‘Ectoparasites’, *Status of Birds in the Hudderfield Area 1959-1974, Annual Report 1975*, pp. 9–10.

Denton, M.L. (1976) ‘Ectoparasites’, *Huddersfield Birdwatchers’ Club Annual Report 1976*, pp. 40–42.

Denton, M.L. (1977) ‘Ectoparasites’, *HUDDERSFIELD BIRDWATCHERS ’ CLUB ANNUAL REPORT 1977*, p. 42. Available at: http://www.huddersfieldbirdwatchersclub.co.uk/annual-reports/4559882266.

Denton, M.L. (1978) ‘Ectoparasites’, *Birds in Huddersfield*, p. 59.

Denton, M.L. (1979) ‘Ectoparasites’, *Birds in Huddersfield 1979*, p. 50.

Denton, M.L. (1980) ‘Ectoparasites’, *Birds in Huddersfield*, p. 54.

Edwards, A.R. (1952) ‘Flatflies taken in the Laboratory during 1951’, *Fair Isle Bird Observatory Annual Report 1951* [Preprint], (37–38). Available at: http://www.fairislebirdobs.co.uk/annual_reports.html.

Edwards, R. (1951) ‘Report on Bird Ectoparasites’, *Fair Isle Bird Observatory Annual Report 1950*, 162, p. 21. Available at: http://www.fairislebirdobs.co.uk/annual_reports.html.

Emley, D.W. (1992) *Staffordshire Flies, a provisional list*. Available at: https://www.google.com/url?sa=t&rct=j&q=&esrc=s&source=web&cd=&ved=2ahUKEwjPxK-sx57uAhUfQxUIHTQOATwQFjAAegQIBhAC&url=http%3A%2F%2Fwww.staffs-ecology.org.uk%2Fhtml2015%2Fimages%2F6%2F67%2FSER015_Staffordshire_Flies_-_A_Provisional_List.pdf&usg=AOvVaw0cBw-0.

Ennion, E.A.R. (1955) ‘Flat-flies collected at Monkhouse Bird Observatory, Northumberland’, *Fair Isle Bird Observatory Bulletin*, 2(7), p. 320. Available at: http://www.fairislebirdobs.co.uk/annual_reports.html.

Gibson, W., Pilkington, J.G. and Pemberton, J.M. (2010) ‘Trypanosoma melophagium from the sheep ked Melophagus ovinus on the island of St Kilda’, *Parasitology*, 137(12), pp. 1799–1804. Available at: https://doi.org/10.1017/S0031182010000752.

Graham, J. *et al.* (1954) ‘Magnificent Frigate Bird in Tiree, Inner Hebrides; A New British Bird’, *British Birds*, 47(2), pp. 58–59.

Hancock, E.G. (1995) ‘insect Records from the West of Scotland in 1993’, *The Glasgow Naturalist*, 22(5), p. 504.

Hancock, E.G. (1996) ‘Parasites on Parsite’, *Glasgow Naturalist*, 23, p. 62.

Harris, D.D. (2009) ‘First record from a parakeet of Ornithomyia avicularia (Linnaeus, 1758) (Diptera, Hippoboscidae)’, *Dipterists Digest*, 16(2), p. 101. Available at: https://www.dipterists.org.uk/digest.

Harrow, M. (2021) ‘Pseudolynchia canariensis (Macquart in Webb & Berthelot) (Diptera, Hippoboscidae) new to Britain’, *Dipterists Digest*, 28(2), p. 166.

Hill, D.S. (1962) ‘A STUDY OF THE DISTRIBUTION AND HOST PREFERENCES OF THREE SPECIES OF ORNITHOMYIA (DIPTERA: HIPPOBOSCIDAE) IN THE BRITISH ISLES’, 37(4–6), pp. 37–48. Available at: https://doi.org/10.1111/j.1365-3032.1962.tb00286.x.

Hill, D.S., Wilson, N. and Corbet, G.B. (1967) ‘Mites Associated With British Species of Ornithomya (Diptera: Hippoboscidae)’, *Journal of Medical Entomology*, 4(2), pp. 102–122. Available at: https://doi.org/10.1093/jmedent/4.2.102.

Hutson, A.M. (1981) ‘The population of the louse-fly, Crataerina pallida (Diptera, Hippoboscidae) on the European Swift, Apus apus (Aves, Apodidae)’, *J. Zoo., Lond.*, 194, pp. 305–316.

Hutson, A.M. (1984) ‘Keds, Flat-flies and Bat-Flies: Diptera, Hippoboscidae and Nycteribiidae’, *Handbooks for the Identification of British Insects*, 10(7), p. 40. Available at: https://www.royensoc.co.uk/sites/default/files/Vol10_Part07_Hutson.pdf.

Johnson, C. (2020) *Species List of the Diptera of the Outer Hebrides (Revised January 2020)*. Available at: https://www.ohbr.org.uk/documents/checklists/Diptera Checklist.pdf.

Kettle, D.S. and Utsi, M.N.P. (1955) ‘Hypoderma diana (Diptera, Oestridae) and Lipoptena cervi (Diptera, Hippoboscidae) as parasites of reindeer (Rangifer tarandus) in Scotland with notes on the second-stage larva of Hypoderma diana’, *Parasitology*, 45(1–2), pp. 116–120. Available at: https://doi.org/10.1017/S0031182000027487.

Lane, R.P. (1978) ‘The Diptera (two-winged flies) of Lundy Island’, *Report ot the Lundy Field Society*, pp. 15–31.

Lloyd-Evans, L. (1967) ‘Bird Parasites’, *Rye Meads Ringing Group Report*, 4, pp. 14–21.

Macdonald, M. (2022) ‘The deer ked Lipoptena cervi (Linnaeus) (Diptera , Hippoboscidae) in Highland , north Scotland’, *Dipterists Digest*, 29, pp. 206–210.

O’Connor, J.P. and Sleeman, D.P. (1987) ‘A Review of the Irish Hippoboscidae (Insecta:Diptera)’, *Irish Naturalists’ Journal*, 22(6 (April)), pp. 236–239.

Palmer, C.J. (1987) ‘Pseudolychnia garzettae Rondani (Dipt., Hippoboscidae), an unrecognized addition to the British List.’, *Entomologist’s monthly Magazine*, 123, p. 234.

Sellers, R.M. and Redgate, N.D. (1992) ‘Some recent records of the grouse louse-fly, Ornithomya chloropus Bergroth (Diptera: Hippoboscidae) from Caithness’, *Entomologist’s Gazette*, 43(4), p. 274.

Smart, J. (1939) ‘Hippoboscidae’, in F.F. Edwards and H. Oldroyd (eds) *Blood-sucking flies*. England: Printed by order of the Trustees of the British Museum, pp. 118–124. Available at: https://babel.hathitrust.org/cgi/pt?id=umn.319510004583588&view=1up&seq=7&skin=2021.

SMART, J. (1945) ‘Ked-Flies’, *Nature*, 155(3926), pp. 123–123. Available at: https://doi.org/10.1038/155123a0.

Smiddy, P. (1997) ‘Louse-flies (Diptera: Hippoboscidae) from the Irish nests of house martins.’, *Irish Naturalists’ Journal*, 25(10), pp. 374–377.

Smiddy, P. and Sleeman, D.P. (2004) ‘Recent Records of Louse-Flies (Diptera: Hippoboscidae) Taken in Ireland’, *Irish Naturalists’ Journal*, 27(10), pp. 403–404.

Stansfield, G. (1954) ‘Collection of Flat-flies on Skokholm Island’, *Skokholm Bird Observatory Report*, pp. 19–22.

Stansfield, G. (1955) ‘Flat-flies and Fleas from Skokholm Birds, 1955’, *Skokholm Bird Observatory Report*, p. 26.

Stansfield, Geoffrey (1955) ‘Flat-fly infestation in the Pipits and Wheatears of Stokholm in 1954’, *Fair Isle Bird Observatory Bulletin*, 2(7), pp. 318–320. Available at: http://www.fairislebirdobs.co.uk/annual_reports.html.

Summers, R.W. (1975) ‘On the ecology of Crataerina hirundinis (Diptera: Hippoboscidae) in Scotland’, *Journal of Zoology, London*, 175, pp. 557–570. Available at: https://doi.org/10.1111/j.1469-7998.1975.tb01417.x.

Theobald, F. V. (1896) *Parasitic diseases of poultry*, *Parasitic diseases of poultry*. London: Gurney. Available at: https://doi.org/10.5962/bhl.title.43848.

Thompson, G.B. (1952) ‘Ectoparasites’, *Gibraltar Point Report*, p. 30.

Thompson, G.B. (1953) ‘Contributions toward a study of the ectoparasites of British birds and mammals.—No. 1’, *Annals and Magazine of Natural History*, 6(66), pp. 401–425. Available at: https://doi.org/10.1080/00222935308654440.

Thompson, G.B. (1954a) ‘LIX.—Contributions toward a study of the ectoparasites of British birds and mammals.—No. 3’, *Annals and Magazine of Natural History*, 7(78), pp. 438–447. Available at: https://doi.org/10.1080/00222935408656061.

Thompson, G.B. (1954b) ‘V.—Contributions toward a study of the ectoparasites of British birds and mammals.—No. 2’, *Annals and Magazine of Natural History*, 7(73), pp. 17–39. Available at: https://doi.org/10.1080/00222935408651687.

Thompson, G.B. (1955a) ‘CVI.—Contributions towards a study of the ectoparasites of British birds and mammals.—No. 6’, *Annals and Magazine of Natural History*, 8(96), pp. 917–927. Available at: https://doi.org/10.1080/00222935508655712.

Thompson, G.B. (1955b) ‘II.—Contributions toward a study of the ectoparasites of British birds and mammals—No. 4’, *Annals and Magazine of Natural History*, 8(85), pp. 25–35. Available at: https://doi.org/10.1080/00222935508651820.

Thompson, G.B. (1955c) ‘LXXXIX.—Contributions toward a study of the Ectoparasites of British birds and Mammals.—No. 5’, *Annals and Magazine of Natural History*, 8(94), pp. 724–730. Available at: https://doi.org/10.1080/00222935508655693.

Thompson, G.B. (1964) ‘Bird ectoparasites collected at Portland Bird Observatory’, *Portland Bird Observatory Report*, p. 29.

Thompson, G.B. (1968) ‘Ornithophila metallica (Schiner) (Diptera: Hippoboscidae) A species of flat-fly new to the British List’, *Bardsey Observatory Report 1967*, pp. 46–47.

Thompson, G.B. and Beaumont, H.E. (1968) ‘Some interesting Records of Flat-flies (Diptera: Hippoboscidae) from Hirundinidae & Apodidae’, in *The Naturalist*. https://www.biodiversitylibrary.org/page/50236244#page/7/mode/1up, pp. 111–114.

Turner, C.R. and Mann, D.J. (2004) ‘Recent Observations of Hippobosca Equina L . ( Diptera : Hippoboscidae ) in South Devon’, *Br. J. Ent. Nat. Hist.*, 17(1274), pp. 1–4. Available at: https://www.researchgate.net/publication/285056258_Recent_observations_of_Hippobosca_equina_L_Diptera_Hippoboscidae_in_south_Devon.

Waine, J. (1997) ‘Report on causes of death amongst avian corpses found on Fair Isle, 1995-6’, *Fair Isle Bird Observatory Report for 1996*, pp. 15–18. Available at: http://www.fairislebirdobs.co.uk/annual_reports.html.

Waine, J. (1999) ‘Report on the causes of deaths in birds on Fair Isle 1998’, *Fair Isle Bird Observatory Report for 1998*, pp. 21–26. Available at: http://www.fairislebirdobs.co.uk/annual_reports.html.

Waine, J. (2000) ‘Report on the causes of Death in wild Bird on Fair isle in 1999’, *Fair Isle Bird Observatory Report for 1999*, pp. 16–18. Available at: http://www.fairislebirdobs.co.uk/annual_reports.html.

Waine, J. (2001) ‘Report on causes of death in wild birds on Fair Isle in 2000’, *Fair Isle Bird Observatory Report for 2000*, pp. 14–16. Available at: http://www.fairislebirdobs.co.uk/annual_reports.html.

Waine, J. (2002) ‘Update on Survey of Diseases and Causes of Death in Wild Birds’, *Fair Isle Bird Observatory Report for 2001*, pp. 16–20. Available at: https://doi.org/10.1038/162989a0.

Waine, J.C. (1993) ‘Disease States and Causes of Death in Wild Birds’, *Fair Isle Bird Observatory Annual Report for 1992*, pp. 69–70. Available at: http://www.fairislebirdobs.co.uk/annual_reports.html.

Waine, J.C. (2004) ‘Report on Investigations into Causes of Death & Disease States in Casualty Birds Found on Fair Isle’, *Fair Isle Bird Observatory Report for 2003*, pp. 20–22. Available at: http://www.fairislebirdobs.co.uk/annual_reports.html.

Waine, J.C. (2005) ‘Report on Investigations into Causes of Death & Disease States in Casualty Birds found on Fair Isle’, *Fair Isle Bird Observatory Report for 2004*, pp. 16–18. Available at: http://www.fairislebirdobs.co.uk/annual_reports.html.

Waine, J.C. (2007) ‘Report on investigations into causes of death and disease states in casualty birds 2005 & 2006’, *Fair Isle Bird Observatory Report for 2006*, pp. 31–36. Available at: http://www.fairislebirdobs.co.uk/annual_reports.html.

Waine, J.C. (2008) ‘Report on investigations into causes of death and disease states in casualty birds found on Fair Isle 2006–2007’, *Fair Isle Bird Observatory Report for 2007*, pp. 12–13. Available at: http://www.fairislebirdobs.co.uk/annual_reports.html.

Waine, J.C. (2009) ‘Report on investigations into causes of death and disease states in casualty birds found on Fair Isle 2007–2008’, *Fair Isle Bird Observatory Report for 2008*, pp. 26–28. Available at: http://www.fairislebirdobs.co.uk/annual_reports.html.

Waine, J.C. (2013) ‘Report on investigations into causes of death and diseases states in casualty birds found on Fair Isle 2009 to 2012’, *Fair Isle Bird Observatory Report for 2012*, (64), pp. 121–124. Available at: http://www.fairislebirdobs.co.uk/annual_reports.html.

Waine, J.C. (2014) ‘Investigations into disease states and the causes of bird deaths on Fair Isle; report for 2013’, *Fair Isle Bird Observatory Report for 2013*, (65), pp. 148–150. Available at: http://www.fairislebirdobs.co.uk/annual_reports.html.

Waterson, J. (1910) ‘IX. - Notes on Some Ectoparasites in the Museum, Perth’, *Transactions - Perthshire Society of Natural Science*, pp. 48–49.

Wawman, D.C. (2024) ‘Ornithomya biloba , Pseudolynchia garzettae and Pseudolynchia canariensis (Diptera : Hippoboscidae): three new United Kingdom colonists and potential disease vectors’, *Medical and Veterinary Entomology*, 38(2), pp. 160–171. Available at: https://doi.org/10.1111/mve.12703.

White, G. (1789a) ‘Letter 15 to Daines Barrington, Selborne, July 8th 1773’, in *The Natural History of Selborne*.

White, G. (1789b) ‘Letter 21 to Daines Barrington, Selborne, September 28th 1774’, in *The Natural History of Selborne*.

White, G. (1789c) ‘Letter 52 to Daines Barrington, Selborne, September 9th 1781’, in *The Natural History of Selborne*.

Williamson, K. (1949) ‘Fair Isle Bird Observatory, First Report 1948’, *the Scottish Naturalist*, 19(1), pp. 19–142. Available at: http://www.fairislebirdobs.co.uk/annual_reports.html.

Williamson, K. (1950) ‘Bird Parasites’, *Fair Isle Bird Observatory Annual Report, 1949*, p. 22. Available at: https://doi.org/10.1038/162989a0.

Williamson, K. (1952) ‘Ectoparasites’, *Fair Isle Bird Observatory, Annual Report 1952*, pp. 13–14. Available at: http://www.fairislebirdobs.co.uk/annual_reports.html.

Williamson, K. (1954) ‘Bird Ectoparasites’, *Fair Isle Bird Observatory, Annual Report 1953*, pp. 14–16. Available at: http://www.fairislebirdobs.co.uk/annual_reports.html.
