# Supplementary material for: Citizen science reveals host‐switching in louse flies and keds (Diptera: Hippoboscidae) during a period of anthropogenic change
Source: Med Vet Entomol. 2025 Nov 1;40(2):305–22. doi: 10.1111/mve.70029 (PMC13140014; doi:10.1111/mve.70029)
Supplement: Supplementary file 2 — Data S2. Table of host species and their masses used in the models. [file MVE-40-305-s002.docx]

**S2. Table of host species used in the models, with their masses (in grams), families and merged groupings**

| **Binomial** | **Species** | **Family** | **Order** | **merged Passerines** | **Merged Waders, Passerines, Seabirds** | **Host mass** |
| --- | --- | --- | --- | --- | --- | --- |
| *Branta bernicla* | Brent Goose | Anatidae | Anseriformes | Anatidae | ANAT | 1236 |
| *Branta canadensis* | Canada Goose | Anatidae | Anseriformes | Anatidae | ANAT | 4128 |
| *Branta leucopsis* | Barnacle Goose | Anatidae | Anseriformes | Anatidae | ANAT | 1895 |
| *Anser anser* | Greylag Goose | Anatidae | Anseriformes | Anatidae | ANAT | 3362 |
| *Anser fabalis* | Taiga Bean Goose | Anatidae | Anseriformes | Anatidae | ANAT |  |
| *Anser brachyrhynchus* | Pink-footed Goose | Anatidae | Anseriformes | Anatidae | ANAT | 2547 |
| *Anser albifrons* | White-fronted Goose | Anatidae | Anseriformes | Anatidae | ANAT | 2542 |
| *Cygnus olor* | Mute Swan | Anatidae | Anseriformes | Anatidae | ANAT | 9605 |
| *Cygnus columbianus* | Bewick's Swan | Anatidae | Anseriformes | Anatidae | ANAT | 6048 |
| *Cygnus cygnus* | Whooper Swan | Anatidae | Anseriformes | Anatidae | ANAT | 8893 |
| *Alopochen aegyptiaca* | Egyptian Goose | Anatidae | Anseriformes | Anatidae | ANAT | 2528 |
| *Tadorna tadorna* | Shelduck | Anatidae | Anseriformes | Anatidae | ANAT | 1220 |
| *Aix galericulata* | Mandarin Duck | Anatidae | Anseriformes | Anatidae | ANAT | 623 |
| *Spatula querquedula* | Garganey | Anatidae | Anseriformes | Anatidae | ANAT | 353 |
| *Spatula clypeata* | Shoveler | Anatidae | Anseriformes | Anatidae | ANAT | 639 |
| *Mareca strepera* | Gadwall | Anatidae | Anseriformes | Anatidae | ANAT | 803 |
| *Mareca penelope* | Wigeon | Anatidae | Anseriformes | Anatidae | ANAT | 740 |
| *Anas platyrhynchos* | Mallard | Anatidae | Anseriformes | Anatidae | ANAT | 1211 |
| *Anas acuta* | Pintail | Anatidae | Anseriformes | Anatidae | ANAT | 938 |
| *Anas crecca* | Teal | Anatidae | Anseriformes | Anatidae | ANAT | 321 |
| *Aythya ferina* | Pochard | Anatidae | Anseriformes | Anatidae | ANAT | 1065 |
| *Aythya fuligula* | Tufted Duck | Anatidae | Anseriformes | Anatidae | ANAT | 797 |
| *Aythya marila* | Scaup | Anatidae | Anseriformes | Anatidae | ANAT |  |
| *Somateria mollissima* | Eider | Anatidae | Anseriformes | Anatidae | ANAT | 1878 |
| *Melanitta fusca* | Velvet Scoter | Anatidae | Anseriformes | Anatidae | ANAT |  |
| *Melanitta nigra* | Common Scoter | Anatidae | Anseriformes | Anatidae | ANAT |  |
| *Clangula hyemalis* | Long-tailed Duck | Anatidae | Anseriformes | Anatidae | ANAT |  |
| *Bucephala clangula* | Goldeneye | Anatidae | Anseriformes | Anatidae | ANAT | 679 |
| *Mergellus albellus* | Smew | Anatidae | Anseriformes | Anatidae | ANAT |  |
| *Mergus merganser* | Goosander | Anatidae | Anseriformes | Anatidae | ANAT | 1214 |
| *Mergus serrator* | Red-breasted Merganser | Anatidae | Anseriformes | Anatidae | ANAT |  |
| *Oxyura jamaicensis* | Ruddy Duck | Anatidae | Anseriformes | Anatidae | ANAT |  |
| *Tetrao urogallus* | Capercaillie | Phasianidae | Galliformes | Phasianidae | Phasianidae | 3150 |
| *Lyrurus tetrix* | Black Grouse | Phasianidae | Galliformes | Phasianidae | Phasianidae | 1050 |
| *Lagopus muta* | Ptarmigan | Phasianidae | Galliformes | Phasianidae | Phasianidae | 450 |
| *Lagopus lagopus* | Red Grouse | Phasianidae | Galliformes | Phasianidae | Phasianidae | 600 |
| *Alectoris rufa* | Red-legged Partridge | Phasianidae | Galliformes | Phasianidae | Phasianidae | 450 |
| *Perdix perdix* | Grey Partridge | Phasianidae | Galliformes | Phasianidae | Phasianidae | 414 |
| *Coturnix coturnix* | Quail | Phasianidae | Galliformes | Phasianidae | Phasianidae | 119 |
| *Phasianus colchicus* | Pheasant | Phasianidae | Galliformes | Phasianidae | Phasianidae | 1190 |
| *Chrysolophus pictus* | Golden Pheasant | Phasianidae | Galliformes | Phasianidae | Phasianidae |  |
| *Chrysolophus amherstiae* | Lady Amherst's Pheasant | Phasianidae | Galliformes | Phasianidae | Phasianidae |  |
| *Gallus gallus domesticus* | Chicken | Phasianidae | Galliformes | Phasianidae | Phasianidae | 2000 |
| *Caprimulgus europaeus* | Nightjar | Caprimulgidae | Caprimulgiformes | Caprimulgidae | Caprimulgidae | 73 |
| *Apus apus* | Swift | Apodidae | Apodiformes | Apodidae | Apodidae | 40 |
| *Cuculus canorus* | Cuckoo | Cuculidae | Cucliformes | Cuculidae | Cuculidae | 115 |
| *Columba livia* | Rock Dove | Columbidae | Columbiformes | Columbidae | Columbidae | 351 |
| *Columba livia domestica* | feral pigeon | Columbidae | Columbiformes | Columbidae | Columbidae | 300 |
| *Columba oenas* | Stock Dove | Columbidae | Columbiformes | Columbidae | Columbidae | 327 |
| *Columba palumbus* | Woodpigeon | Columbidae | Columbiformes | Columbidae | Columbidae | 515 |
| *Streptopelia turtur* | Turtle Dove | Columbidae | Columbiformes | Columbidae | Columbidae | 156 |
| *Streptopelia decaocto* | Collared Dove | Columbidae | Columbiformes | Columbidae | Columbidae | 204 |
| *Rallus aquaticus* | Water Rail | Rallidae | Gruiformes | Rallidae | Rallidae | 125 |
| *Crex crex* | Corncrake | Rallidae | Gruiformes | Rallidae | Rallidae | 168 |
| *Porzana porzana* | Spotted Crake | Rallidae | Gruiformes | Rallidae | Rallidae | 85.5 |
| *Gallinula chloropus* | Moorhen | Rallidae | Gruiformes | Rallidae | Rallidae | 364 |
| *Fulica atra* | Coot | Rallidae | Gruiformes | Rallidae | Rallidae | 868 |
| *Grus grus* | Crane | Gruidae | Gruiformes | Gruidae | GRUI |  |
| *Tachybaptus ruficollis* | Little Grebe | Podicipedidae | Podicipediformes | Podicipedidae | PODI | 220 |
| *Podiceps grisegena* | Red-necked Grebe | Podicipedidae | Podicipediformes | Podicipedidae | PODI |  |
| *Podiceps cristatus* | Great Crested Grebe | Podicipedidae | Podicipediformes | Podicipedidae | PODI | 920 |
| *Podiceps auritus* | Slavonian Grebe | Podicipedidae | Podicipediformes | Podicipedidae | PODI | 446 |
| *Podiceps nigricollis* | Black-necked Grebe | Podicipedidae | Podicipediformes | Podicipedidae | PODI |  |
| *Burhinus oedicnemus* | Stone-curlew | Burhinidae | Charadriformes | Burhinidae | Waders | 431 |
| *Haematopus ostralegus* | Oystercatcher | Haematopodidae | Charadriformes | Haematopodidae | Waders | 538 |
| *Recurvirostra avosetta* | Avocet | Recurvirostridae | Charadriiformes | Recurvirostridae | Waders | 340 |
| *Vanellus vanellus* | Lapwing | Charadriidae | Charadriiformes | Charadriidae | Waders | 236 |
| *Pluvialis apricaria* | Golden Plover | Charadriidae | Charadriiformes | Charadriidae | Waders | 192 |
| *Pluvialis squatarola* | Grey Plover | Charadriidae | Charadriiformes | Charadriidae | Waders | 226 |
| *Charadrius hiaticula* | Ringed Plover | Charadriidae | Charadriiformes | Charadriidae | Waders | 65 |
| *Charadrius dubius* | Little Ringed Plover | Charadriidae | Charadriiformes | Charadriidae | Waders | 37 |
| *Charadrius morinellus* | Dotterel | Charadriidae | Charadriiformes | Charadriidae | Waders | 115 |
| *Numenius phaeopus* | Whimbrel | Scolopacidae | Charadriiformes | Scolopacidae | Waders | 440 |
| *Numenius arquata* | Curlew | Scolopacidae | Charadriiformes | Scolopacidae | Waders | 786 |
| *Limosa lapponica* | Bar-tailed Godwit | Scolopacidae | Charadriiformes | Scolopacidae | Waders | 293 |
| *Limosa limosa* | Black-tailed Godwit | Scolopacidae | Charadriiformes | Scolopacidae | Waders | 301 |
| *Arenaria interpres* | Turnstone | Scolopacidae | Charadriiformes | Scolopacidae | Waders | 107 |
| *Calidris canutus* | Knot | Scolopacidae | Charadriiformes | Scolopacidae | Waders | 137 |
| *Calidris pugnax* | Ruff | Scolopacidae | Charadriiformes | Scolopacidae | Waders | 164 |
| *Calidris ferruginea* | Curlew Sandpiper | Scolopacidae | Charadriiformes | Scolopacidae | Waders | 66 |
| *Calidris temminckii* | Temminck's Stint | Scolopacidae | Charadriiformes | Scolopacidae | Waders |  |
| *Calidris alba* | Sanderling | Scolopacidae | Charadriiformes | Scolopacidae | Waders | 56 |
| *Calidris alpina* | Dunlin | Scolopacidae | Charadriiformes | Scolopacidae | Waders | 49 |
| *Calidris maritima* | Purple Sandpiper | Scolopacidae | Charadriiformes | Scolopacidae | Waders | 70 |
| *Calidris minuta* | Little Stint | Scolopacidae | Charadriiformes | Scolopacidae | Waders | 27 |
| *Scolopax rusticola* | Woodcock | Scolopacidae | Charadriiformes | Scolopacidae | Waders | 325 |
| *Lymnocryptes minimus* | Jack Snipe | Scolopacidae | Charadriiformes | Scolopacidae | Waders | 59 |
| *Gallinago media* | Great Snipe | Scolopacidae | Charadriiformes | Scolopacidae | Waders | 175 |
| *Gallinago gallinago* | Snipe | Scolopacidae | Charadriiformes | Scolopacidae | Waders | 109 |
| *Phalaropus lobatus* | Red-necked Phalarope | Scolopacidae | Charadriiformes | Scolopacidae | Waders |  |
| *Phalaropus fulicarius* | Grey Phalarope | Scolopacidae | Charadriiformes | Scolopacidae | Waders |  |
| *Actitis hypoleucos* | Common Sandpiper | Scolopacidae | Charadriiformes | Scolopacidae | Waders | 57 |
| *Tringa ochropus* | Green Sandpiper | Scolopacidae | Charadriiformes | Scolopacidae | Waders | 83 |
| *Tringa totanus* | Redshank | Scolopacidae | Charadriiformes | Scolopacidae | Waders | 150 |
| *Tringa glareola* | Wood Sandpiper | Scolopacidae | Charadriiformes | Scolopacidae | Waders | 55.7 |
| *Tringa erythropus* | Spotted Redshank | Scolopacidae | Charadriiformes | Scolopacidae | Waders | 155 |
| *Tringa nebularia* | Greenshank | Scolopacidae | Charadriiformes | Scolopacidae | Waders | 191 |
| *Rissa tridactyla* | Kittiwake | Laridae | Charadriiformes | Laridae | Laridae | 365 |
| *Chroicocephalus ridibundus* | Black-headed Gull | Laridae | Charadriiformes | Laridae | Laridae | 289 |
| *Hydrocoloeus minutus* | Little Gull | Laridae | Charadriiformes | Laridae | Laridae | 110 |
| *Ichthyaetus melanocephalus* | Mediterranean Gull | Laridae | Charadriiformes | Laridae | Laridae | 330 |
| *Larus canus* | Common Gull | Laridae | Charadriiformes | Laridae | Laridae | 405 |
| *Larus marinus* | Great Black-backed Gull | Laridae | Charadriiformes | Laridae | Laridae | 1538 |
| *Larus hyperboreus* | Glaucous Gull | Laridae | Charadriiformes | Laridae | Laridae |  |
| *Larus glaucoides* | Iceland Gull | Laridae | Charadriiformes | Laridae | Laridae |  |
| *Larus argentatus* | Herring Gull | Laridae | Charadriiformes | Laridae | Laridae | 940 |
| *Larus michahellis* | Yellow-legged Gull | Laridae | Charadriiformes | Laridae | Laridae |  |
| *Larus fuscus* | Lesser Black-backed Gull | Laridae | Charadriiformes | Laridae | Laridae | 822 |
| *Hydroprogne caspia* | Caspian Tern | Laridae | Charadriiformes | Laridae | Laridae |  |
| *Thalasseus sandvicensis* | Sandwich Tern | Laridae | Charadriiformes | Laridae | Laridae | 236 |
| *Sternula albifrons* | Little Tern | Laridae | Charadriiformes | Laridae | Laridae | 55 |
| *Sterna dougallii* | Roseate Tern | Laridae | Charadriiformes | Laridae | Laridae | 115 |
| *Sterna hirundo* | Common Tern | Laridae | Charadriiformes | Laridae | Laridae | 129 |
| *Sterna paradisaea* | Arctic Tern | Laridae | Charadriiformes | Laridae | Laridae | 272 |
| *Chlidonias niger* | Black Tern | Laridae | Charadriiformes | Laridae | Laridae |  |
| *Stercorarius skua* | Great Skua | Stercorariidae | Charadriiformes | STE | Stercorariidae | 1331 |
| *Stercorarius parasiticus* | Arctic Skua | Stercorariidae | Charadriiformes | STE | Stercorariidae | 420 |
| *Uria aalge* | Common Guillemot | Alcidae | Charadriiformes | Alcidae | SEAB | 923 |
| *Alca torda* | Razorbill | Alcidae | Charadriiformes | Alcidae | SEAB | 614 |
| *Cepphus grylle* | Black Guillemot | Alcidae | Charadriiformes | Alcidae | SEAB | 406 |
| *Fratercula arctica* | Puffin | Alcidae | Charadriiformes | Alcidae | SEAB | 383 |
| *Gavia stellata* | Red-throated Diver | Phaethontidae | Phaethonidae | PHAE | SEAB | 1665 |
| *Gavia arctica* | Black-throated Diver | Phaethontidae | Phaethonidae | PHAE | SEAB |  |
| *Gavia immer* | Great Northern Diver | Phaethontidae | Phaethonidae | PHAE | SEAB |  |
| *Hydrobates pelagicus* | Storm Petrel | Hydrobatidae | Procellariformes | Hydrobatidae | SEAB | 25 |
| *Oceanodroma leucorhoa* | Leach's Petrel | Hydrobatidae | Procellariformes | Hydrobatidae | SEAB | 43 |
| *Fulmarus glacialis* | Fulmar | Procellariidae | Procellariformes | Procellariidae | SEAB | 779 |
| *Puffinus puffinus* | Manx Shearwater | Procellariidae | Procellariformes | Procellariidae | SEAB | 392 |
| *Morus bassanus* | Gannet | Sulidae | Suliformes | Sulidae | SEAB | 2990 |
| *Phalacrocorax carbo* | Cormorant | Phalacrocoracidae | Phalacrocoracidae | Phalacrocoracidae | SEAB | 2702 |
| *Phalacrocorax aristotelis* | Shag | Phalacrocoracidae | Phalacrocoracidae | Phalacrocoracidae | SEAB | 1780 |
| *Plegadis falcinellus* | Glossy Ibis | Theskiornithidae | Pelecaniformes | Theskiornithidae | THES |  |
| *Platalea leucorodia* | Spoonbill | Theskiornithidae | Pelecaniformes | Theskiornithidae | THES |  |
| *Botaurus stellaris* | Bittern | Ardeidae | Pelecaniformes | Ardeidae | Ardeidae | 1250 |
| *Ixobrychus minutus* | Little Bittern | Ardeidae | Pelecaniformes | Ardeidae | Ardeidae |  |
| *Bubulcus ibis* | Cattle Egret | Ardeidae | Pelecaniformes | Ardeidae | Ardeidae |  |
| *Ardea cinerea* | Grey Heron | Ardeidae | Pelecaniformes | Ardeidae | Ardeidae | 1675 |
| *Ardea purpurea* | Purple Heron | Ardeidae | Pelecaniformes | Ardeidae | Ardeidae | 1250 |
| *Ardea alba* | Great White Egret | Ardeidae | Pelecaniformes | Ardeidae | Ardeidae |  |
| *Egretta garzetta* | Little Egret | Ardeidae | Pelecaniformes | Ardeidae | Ardeidae | 488 |
| *Pandion haliaetus* | Osprey | Pandionidae | Acciptriformes | PAND | Pandionidae | 1372.5 |
| *Pernis apivorus* | Honey-buzzard | Accipitridae | Acciptriformes | Accipitridae | Accipitridae |  |
| *Aquila chrysaetos* | Golden Eagle | Accipitridae | Acciptriformes | Accipitridae | Accipitridae | 4550 |
| *Accipiter nisus* | Sparrowhawk | Accipitridae | Acciptriformes | Accipitridae | Accipitridae | 181 |
| *Accipiter gentilis* | Goshawk | Accipitridae | Acciptriformes | Accipitridae | Accipitridae | 1040 |
| *Circus aeruginosus* | Marsh Harrier | Accipitridae | Acciptriformes | Accipitridae | Accipitridae |  |
| *Circus cyaneus* | Hen Harrier | Accipitridae | Acciptriformes | Accipitridae | Accipitridae | 490 |
| *Circus pygargus* | Montagu's Harrier | Accipitridae | Acciptriformes | Accipitridae | Accipitridae |  |
| *Milvus milvus* | Red Kite | Accipitridae | Acciptriformes | Accipitridae | Accipitridae | 929 |
| *Haliaeetus albicilla* | White-tailed Eagle | Accipitridae | Acciptriformes | Accipitridae | Accipitridae |  |
| *Buteo lagopus* | Rough-legged Buzzard | Accipitridae | Acciptriformes | Accipitridae | Accipitridae |  |
| *Buteo buteo* | Buzzard | Accipitridae | Acciptriformes | Accipitridae | Accipitridae | 917 |
| *Tyto alba* | Barn Owl | Tytonidae | Strigiformes | Tytonidae | Tytonidae | 339 |
| *Athene noctua* | Little Owl | Strigidae | Strigiformes | Strigidae | Strigidae | 190 |
| *Asio otus* | Long-eared Owl | Strigidae | Strigiformes | Strigidae | Strigidae | 288 |
| *Asio flammeus* | Short-eared Owl | Strigidae | Strigiformes | Strigidae | Strigidae | 358 |
| *Strix aluco* | Tawny Owl | Strigidae | Strigiformes | Strigidae | Strigidae | 481 |
| *Upupa epops* | Hoopoe | Upupidae | Coraciiformes | Upupidae | UPUP |  |
| *Alcedo atthis* | Kingfisher | Alcedinidae | Coraciiformes | Alcedinidae | ALCE | 39 |
| *Merops apiaster* | Bee-eater | Meropidae | Coraciiformes | Meropidae | MERO |  |
| *Jynx torquilla* | Wryneck | Picidae | Coraciiformes | Picidae | Picidae | 33 |
| *Dryobates minor* | Lesser Spotted Woodpecker | Picidae | Coraciiformes | Picidae | Picidae | 20 |
| *Dendrocopos major* | Great Spotted Woodpecker | Picidae | Coraciiformes | Picidae | Picidae | 78 |
| *Picus viridis* | Green Woodpecker | Picidae | Coraciiformes | Picidae | Picidae | 189 |
| *Falco tinnunculus* | Kestrel | Falconidae | Falconiformes | Falconidae | Falconidae | 204 |
| *Falco vespertinus* | Red-footed Falcon | Falconidae | Falconiformes | Falconidae | Falconidae |  |
| *Falco columbarius* | Merlin | Falconidae | Falconiformes | Falconidae | Falconidae | 213 |
| *Falco subbuteo* | Hobby | Falconidae | Falconiformes | Falconidae | Falconidae | 257 |
| *Falco peregrinus* | Peregrine | Falconidae | Falconiformes | Falconidae | Falconidae | 1063 |
| *Psittacula krameri* | Ring-necked Parakeet | Psittaculidae | Psittaciformes | Psittaculidae | Psittaculidae | 142 |
| *Lanius collurio* | Red-backed Shrike | Laniidae | Passeriformes | Passeriformes | Passeriformes | 28 |
| *Lanius excubitor* | Great Grey Shrike | Laniidae | Passeriformes | Passeriformes | Passeriformes | 62.7 |
| *Oriolus oriolus* | Golden Oriole | Oriolidae | Passeriformes | Passeriformes | Passeriformes | 67 |
| *Garrulus glandarius* | Jay | Corvidae | Passeriformes | Passeriformes | Passeriformes | 167 |
| *Pica pica* | Magpie | Corvidae | Passeriformes | Passeriformes | Passeriformes | 210 |
| *Pyrrhocorax pyrrhocorax* | Chough | Corvidae | Passeriformes | Passeriformes | Passeriformes | 325 |
| *Coloeus monedula* | Jackdaw | Corvidae | Passeriformes | Passeriformes | Passeriformes | 230 |
| *Corvus frugilegus* | Rook | Corvidae | Passeriformes | Passeriformes | Passeriformes | 440 |
| *Corvus corone* | Carrion Crow | Corvidae | Passeriformes | Passeriformes | Passeriformes | 511 |
| *Corvus cornix* | Hooded Crow | Corvidae | Passeriformes | Passeriformes | Passeriformes | 529 |
| *Corvus corax* | Raven | Corvidae | Passeriformes | Passeriformes | Passeriformes | 1222.5 |
| *Bombycilla garrulus* | Waxwing | Bombycillidae | Passeriformes | Passeriformes | Passeriformes | 58 |
| *Periparus ater* | Coal Tit | Paridae | Passeriformes | Passeriformes | Passeriformes | 9 |
| *Lophophanes cristatus* | Crested Tit | Paridae | Passeriformes | Passeriformes | Passeriformes | 10 |
| *Poecile palustris* | Marsh Tit | Paridae | Passeriformes | Passeriformes | Passeriformes | 10 |
| *Poecile montanus* | Willow Tit | Paridae | Passeriformes | Passeriformes | Passeriformes | 10 |
| *Cyanistes caeruleus* | Blue Tit | Paridae | Passeriformes | Passeriformes | Passeriformes | 10 |
| *Parus major* | Great Tit | Paridae | Passeriformes | Passeriformes | Passeriformes | 18 |
| *Panurus biarmicus* | Bearded Tit | Panuridae | Passeriformes | Passeriformes | Passeriformes | 15 |
| *Lullula arborea* | Woodlark | Alaudidae | Passeriformes | Passeriformes | Passeriformes | 29 |
| *Alauda arvensis* | Skylark | Alaudidae | Passeriformes | Passeriformes | Passeriformes | 38 |
| *Eremophila alpestris* | Shore Lark | Alaudidae | Passeriformes | Passeriformes | Passeriformes | 35 |
| *Riparia riparia* | Sand Martin | Hirundinidae | Passeriformes | Passeriformes | Passeriformes | 13 |
| *Hirundo rustica* | Swallow | Hirundinidae | Passeriformes | Passeriformes | Passeriformes | 19 |
| *Delichon urbicum* | House Martin | Hirundinidae | Passeriformes | Passeriformes | Passeriformes | 17 |
| *Cettia cetti* | Cetti's Warbler | Cettiidae | Passeriformes | Passeriformes | Passeriformes | 13 |
| *Aegithalos caudatus* | Long-tailed Tit | Aegithalidae | Passeriformes | Passeriformes | Passeriformes | 7 |
| *Phylloscopus sibilatrix* | Wood Warbler | Phylloscopidae | Passeriformes | Passeriformes | Passeriformes | 9 |
| *Phylloscopus trochilus* | Willow Warbler | Phylloscopidae | Passeriformes | Passeriformes | Passeriformes | 8 |
| *Phylloscopus collybita* | Chiffchaff | Phylloscopidae | Passeriformes | Passeriformes | Passeriformes | 7 |
| *Acrocephalus paludicola* | Aquatic Warbler | Acrocephalidae | Passeriformes | Passeriformes | Passeriformes | 12.9 |
| *Acrocephalus schoenobaenus* | Sedge Warbler | Acrocephalidae | Passeriformes | Passeriformes | Passeriformes | 11 |
| *Acrocephalus agricola* | Paddyfield Warbler | Acrocephalidae | Passeriformes | Passeriformes | Passeriformes |  |
| *Acrocephalus scirpaceus* | Reed Warbler | Acrocephalidae | Passeriformes | Passeriformes | Passeriformes | 11 |
| *Acrocephalus palustris* | Marsh Warbler | Acrocephalidae | Passeriformes | Passeriformes | Passeriformes | 13 |
| *Hippolais icterina* | Icterine Warbler | Acrocephalidae | Passeriformes | Passeriformes | Passeriformes | 13 |
| *Locustella luscinioides* | Savi's Warbler | Locustellidae | Passeriformes | Passeriformes | Passeriformes | 15 |
| *Locustella naevia* | Grasshopper Warbler | Locustellidae | Passeriformes | Passeriformes | Passeriformes | 13 |
| *Sylvia atricapilla* | Blackcap | Sylviidae | Passeriformes | Passeriformes | Passeriformes | 17 |
| *Sylvia borin* | Garden Warbler | Sylviidae | Passeriformes | Passeriformes | Passeriformes | 17 |
| *Curruca nisoria* | Barred Warbler | Sylviidae | Passeriformes | Passeriformes | Passeriformes | 24.3 |
| *Curruca curruca* | Lesser Whitethroat | Sylviidae | Passeriformes | Passeriformes | Passeriformes | 11 |
| *Curruca communis* | Whitethroat | Sylviidae | Passeriformes | Passeriformes | Passeriformes | 13 |
| *Curruca undata* | Dartford Warbler | Sylviidae | Passeriformes | Passeriformes | Passeriformes | 9 |
| *Regulus ignicapilla* | Firecrest | Regulidae | Passeriformes | Passeriformes | Passeriformes | 5 |
| *Regulus regulus* | Goldcrest | Regulidae | Passeriformes | Passeriformes | Passeriformes | 5 |
| *Troglodytes troglodytes* | Wren | Troglodytidae | Passeriformes | Passeriformes | Passeriformes | 9 |
| *Sitta europaea* | Nuthatch | Sittidae | Passeriformes | Passeriformes | Passeriformes | 22 |
| *Certhia familiaris* | Treecreeper | Certhiidae | Passeriformes | Passeriformes | Passeriformes | 8 |
| *Sturnus vulgaris* | Starling | Sturnidae | Passeriformes | Passeriformes | Passeriformes | 83 |
| *Zoothera aurea* | White's Thrush | Turdidae | Passeriformes | Passeriformes | Passeriformes |  |
| *Turdus torquatus* | Ring Ouzel | Turdidae | Passeriformes | Passeriformes | Passeriformes | 105 |
| *Turdus merula* | Blackbird | Turdidae | Passeriformes | Passeriformes | Passeriformes | 101 |
| *Turdus pilaris* | Fieldfare | Turdidae | Passeriformes | Passeriformes | Passeriformes | 108 |
| *Turdus iliacus* | Redwing | Turdidae | Passeriformes | Passeriformes | Passeriformes | 65 |
| *Turdus philomelos* | Song Thrush | Turdidae | Passeriformes | Passeriformes | Passeriformes | 74 |
| *Turdus viscivorus* | Mistle Thrush | Turdidae | Passeriformes | Passeriformes | Passeriformes | 125 |
| *Muscicapa striata* | Spotted Flycatcher | Muscicapidae | Passeriformes | Passeriformes | Passeriformes | 14 |
| *Erithacus rubecula* | Robin | Muscicapidae | Passeriformes | Passeriformes | Passeriformes | 19 |
| *Luscinia svecica* | Bluethroat | Muscicapidae | Passeriformes | Passeriformes | Passeriformes | 16.7 |
| *Luscinia megarhynchos* | Nightingale | Muscicapidae | Passeriformes | Passeriformes | Passeriformes | 21 |
| *Ficedula hypoleuca* | Pied Flycatcher | Muscicapidae | Passeriformes | Passeriformes | Passeriformes | 13 |
| *Phoenicurus ochruros* | Black Redstart | Muscicapidae | Passeriformes | Passeriformes | Passeriformes | 16 |
| *Phoenicurus phoenicurus* | Redstart | Muscicapidae | Passeriformes | Passeriformes | Passeriformes | 14 |
| *Saxicola rubetra* | Whinchat | Muscicapidae | Passeriformes | Passeriformes | Passeriformes | 16 |
| *Saxicola rubicola* | Stonechat | Muscicapidae | Passeriformes | Passeriformes | Passeriformes | 15.4 |
| *Oenanthe oenanthe* | Wheatear | Muscicapidae | Passeriformes | Passeriformes | Passeriformes | 28 |
| *Cinclus cinclus* | Dipper | Cinclidae | Passeriformes | Passeriformes | Passeriformes | 63 |
| *Passer domesticus* | House Sparrow | Passeridae | Passeriformes | Passeriformes | Passeriformes | 27 |
| *Passer montanus* | Tree Sparrow | Passeridae | Passeriformes | Passeriformes | Passeriformes | 21 |
| *Prunella modularis* | Dunnock | Prunellidae | Passeriformes | Passeriformes | Passeriformes | 21 |
| *Motacilla flava* | Yellow Wagtail | Motacillidae | Passeriformes | Passeriformes | Passeriformes | 17 |
| *Motacilla citreola* | Citrine Wagtail | Motacillidae | Passeriformes | Passeriformes | Passeriformes |  |
| *Motacilla cinerea* | Grey Wagtail | Motacillidae | Passeriformes | Passeriformes | Passeriformes | 18 |
| *Motacilla alba* | Pied Wagtail | Motacillidae | Passeriformes | Passeriformes | Passeriformes | 23 |
| *Anthus pratensis* | Meadow Pipit | Motacillidae | Passeriformes | Passeriformes | Passeriformes | 18 |
| *Anthus trivialis* | Tree Pipit | Motacillidae | Passeriformes | Passeriformes | Passeriformes | 21 |
| *Anthus spinoletta* | Water Pipit | Motacillidae | Passeriformes | Passeriformes | Passeriformes | 24 |
| *Anthus petrosus* | Rock Pipit | Motacillidae | Passeriformes | Passeriformes | Passeriformes | 25 |
| *Fringilla coelebs* | Chaffinch | Fringillidae | Passeriformes | Passeriformes | Passeriformes | 21 |
| *Fringilla montifringilla* | Brambling | Fringillidae | Passeriformes | Passeriformes | Passeriformes | 24 |
| *Coccothraustes coccothraustes* | Hawfinch | Fringillidae | Passeriformes | Passeriformes | Passeriformes | 52 |
| *Pyrrhula pyrrhula* | Bullfinch | Fringillidae | Passeriformes | Passeriformes | Passeriformes | 22 |
| *Carpodacus erythrinus* | Common Rosefinch | Fringillidae | Passeriformes | Passeriformes | Passeriformes |  |
| *Chloris chloris* | Greenfinch | Fringillidae | Passeriformes | Passeriformes | Passeriformes | 87 |
| *Linaria flavirostris* | Twite | Fringillidae | Passeriformes | Passeriformes | Passeriformes | 16 |
| *Linaria cannabina* | Linnet | Fringillidae | Passeriformes | Passeriformes | Passeriformes | 18 |
| *Acanthis flammea* | Redpoll | Fringillidae | Passeriformes | Passeriformes | Passeriformes |  |
| *Loxia pytyopsittacus* | Parrot Crossbill | Fringillidae | Passeriformes | Passeriformes | Passeriformes | 50 |
| *Loxia scotica* | Scottish Crossbill | Fringillidae | Passeriformes | Passeriformes | Passeriformes |  |
| *Loxia curvirostra* | Crossbill | Fringillidae | Passeriformes | Passeriformes | Passeriformes | 40 |
| *Carduelis carduelis* | Goldfinch | Fringillidae | Passeriformes | Passeriformes | Passeriformes | 15 |
| *Serinus serinus* | Serin | Fringillidae | Passeriformes | Passeriformes | Passeriformes | 11 |
| *Spinus spinus* | Siskin | Fringillidae | Passeriformes | Passeriformes | Passeriformes | 12 |
| *Calcarius lapponicus* | Lapland Bunting | Calcariidae | Passeriformes | Passeriformes | Passeriformes | 26 |
| *Plectrophenax nivalis* | Snow Bunting | Calcariidae | Passeriformes | Passeriformes | Passeriformes | 33 |
| *Emberiza calandra* | Corn Bunting | Calcariidae | Passeriformes | Passeriformes | Passeriformes | 46 |
| *Emberiza citrinella* | Yellowhammer | Calcariidae | Passeriformes | Passeriformes | Passeriformes | 25 |
| *Emberiza hortulana* | Ortolan Bunting | Calcariidae | Passeriformes | Passeriformes | Passeriformes |  |
| *Emberiza cirlus* | Cirl Bunting | Calcariidae | Passeriformes | Passeriformes | Passeriformes | 24 |
| *Emberiza schoeniclus* | Reed Bunting | Calcariidae | Passeriformes | Passeriformes | Passeriformes | 19 |
| *Rangifer tarandus* | Reindeer | Cervidae | Mammaliaformes | Cervidae | Cervidae | 170 |
| *Dama dama* | Fallow Deer | Cervidae | Mammaliaformes | Cervidae | Cervidae | 65000 |
| *Capreolus capreolus* | Roe Deer | Cervidae | Mammaliaformes | Cervidae | Cervidae | 25 |
| *Cervus nippon* | Sika Deer | Cervidae | Mammaliaformes | Cervidae | Cervidae | 42000 |
| *Cervus elaphus* | Red Deer | Cervidae | Mammaliaformes | Cervidae | Cervidae | 200000 |
| *Ovis aries* | Sheep | Bovidae | Mammaliaformes | Bovidae | Bovidae | 80000 |
| *Bos taurus* | Cattle | Bovidae | Mammaliaformes | Bovidae | Bovidae | 510000 |
| *Equus ferus caballus* | Horse | Equidae | Mammaliaformes | Equidae | Equidae | 450000 |
| *Meles Meles* | Badger | Mustelidae | Mammaliaformes | Mustelidae | Mustelidae | 15000 |
| *Canis familiaris* | Dog | Canidae | Mammaliaformes | Canidae | Canidae | 40000 |
| *Homo sapiens* | Human | Hominidae | Mammaliaformes | Hominidae | Hominidae | 70000 |
