# Supplementary material for: Citizen science reveals host‐switching in louse flies and keds (Diptera: Hippoboscidae) during a period of anthropogenic change
Source: Med Vet Entomol. 2025 Nov 1;40(2):305–22. doi: 10.1111/mve.70029 (PMC13140014; doi:10.1111/mve.70029)
Supplement: Supplementary file 3 — Data S3. Table of all known host‐Hippoboscidae interactions from the United Kingdom, Republic of Ireland and Isle of Man. [file MVE-40-305-s001.docx]

**S3. Table of known host-Hippoboscid interactions from the United Kingdom, Republic of Ireland and Isle of Man.**

The numbers in the columns refer to the references for the original report (listed below), if it is available, or attributed to the paper in which the references can be found. Those listed as “NEW” are interactions first reported in this study.

Unfortunately, it was necessary to exclude many of the earlier published host-parasite interactions because *Ornithomya chloropus* was not recognised as a separate species and records were included with those of *O. fringillina.*

| ***Host British vernacular name*** | Generalists | | | Stenoxenous louse flies | | | | | Vagrant louse flies | | | | | Keds | | | | ***Host scientific name*** |
| --- | --- | --- | --- | --- | --- | --- | --- | --- | --- | --- | --- | --- | --- | --- | --- | --- | --- | --- |
|  | ***Ornithomya avicularia*** | ***Ornithomya chloropus*** | ***Ornithomya fringillina*** | ***Ornithomya biloba*** | ***Crataerina pallida*** | ***Stenepteryx hirundinis*** | ***Pseudolynchia canariensis*** | ***Pseudolynchia garzettae*** | ***Ornithophila gestroi*** | ***Ornithophila metallica*** | ***Icosta ardae*** | ***Icosta minor*** | ***Olfersia spinifera*** | ***Hippobosca longipennis*** | ***Hippobosca equina*** | ***Lipoptena cervi*** | ***Melophagus ovinus*** |  |
| Black Grouse | 2 | 13 | 21 |  |  |  |  |  |  |  |  |  |  |  |  |  |  | *Lyrurus tetrix* |
| Ptarmigan |  | 13 |  |  |  |  |  |  |  |  |  |  |  |  |  |  |  | *Lagopus muta* |
| Red Grouse | 2 | 2 |  |  |  |  |  |  |  |  |  |  |  |  |  |  |  | *Lagopus lagopus* |
| Red-legged Partridge | 21 |  |  |  |  |  |  |  |  |  |  |  |  |  |  |  |  | *Alectoris rufa* |
| Grey Partridge | 2 | 13 | 21 |  |  |  |  |  |  |  |  |  |  |  |  |  |  | *Perdix perdix* |
| Pheasant | 2 |  |  |  |  |  |  |  |  |  |  |  |  |  |  |  |  | *Phasianus colchicus* |
| Chicken | 33 |  |  |  |  |  |  |  |  |  |  |  |  |  |  | 33 |  | *Gallus gallus domesticus* |
| Nightjar | NEW |  |  |  |  |  |  | 34 |  |  |  |  |  |  |  |  |  | *Caprimulgus europaeus* |
| Swift |  |  |  |  | 35 | 1 |  |  |  |  |  |  |  |  |  |  |  | *Apus apus* |
| Cuckoo | 2 | 25 |  |  |  |  |  |  |  |  |  |  |  |  |  |  |  | *Cuculus canorus* |
| Rock Dove | 2 |  | NEW |  |  |  |  |  |  |  |  |  |  |  |  |  |  | *Columba livia* |
| feral pigeon | 2 |  |  |  |  |  | NEW |  |  |  |  |  |  |  |  |  |  | *Columba livia domestica* |
| Stock Dove | NEW | NEW |  |  |  |  |  |  |  |  |  |  |  |  |  |  |  | *Columba oenas* |
| Woodpigeon | 2 |  |  |  |  |  |  |  |  |  |  |  |  |  |  |  |  | *Columba palumbus* |
| Turtle Dove | 2 |  |  |  |  |  |  |  |  |  |  |  |  |  |  |  |  | *Streptopelia turtur* |
| Collared Dove | 2 |  |  |  |  |  |  |  |  |  |  |  |  |  |  |  |  | *Streptopelia decaocto* |
| Water Rail | 27 | 13 |  |  |  |  |  |  |  |  |  |  |  |  |  |  |  | *Rallus aquaticus* |
| Corncrake |  |  | 2 |  |  |  |  |  |  |  |  | 28 |  |  |  |  |  | *Crex crex* |
| Moorhen | 2 | 42 |  |  |  |  |  |  |  |  |  |  |  |  |  |  |  | *Gallinula chloropus* |
| Oystercatcher |  | 40 | NEW |  |  |  |  |  |  |  |  |  |  |  |  |  |  | *Haematopus ostralegus* |
| Lapwing |  | 13 |  |  |  |  |  |  |  |  |  |  |  |  |  |  |  | *Vanellus vanellus* |
| Golden Plover |  | 13 |  |  |  |  |  |  |  |  |  |  |  |  |  |  |  | *Pluvialis apricaria* |
| Ringed Plover | 2 | 40 |  |  |  |  |  |  |  |  |  |  |  |  |  |  |  | *Charadrius hiaticula* |
| Curlew |  | 41 |  |  |  |  |  |  |  |  |  |  |  |  |  |  |  | *Numenius arquata* |
| Black-tailed Godwit |  | NEW |  |  |  |  |  |  |  |  |  |  |  |  |  |  |  | *Limosa limosa* |
| Turnstone |  | NEW |  |  |  |  |  |  |  |  |  |  |  |  |  |  |  | *Arenaria interpres* |
| Dunlin |  | 13 |  |  |  |  |  |  |  |  |  |  |  |  |  |  |  | *Calidris alpina* |
| Woodcock | 13 | 8 |  |  |  |  |  |  |  |  |  |  |  |  |  |  |  | *Scolopax rusticola* |
| Jack Snipe |  | NEW |  |  |  |  |  |  |  |  |  |  |  |  |  |  |  | *Lymnocryptes minimus* |
| Great Snipe |  | NEW |  |  |  |  |  |  |  |  |  |  |  |  |  |  |  | *Gallinago media* |
| Snipe | 2 | 40 |  |  |  |  |  |  |  |  |  |  |  |  |  |  |  | *Gallinago gallinago* |
| Common Sandpiper | NEW | 2 |  |  |  |  |  |  |  |  |  |  |  |  |  |  |  | *Actitis hypoleucos* |
| Green Sandpiper |  | 25 |  |  |  |  |  |  |  |  |  |  |  |  |  |  |  | *Tringa ochropus* |
| Redshank |  | 13 |  |  |  |  |  |  |  |  |  |  |  |  |  |  |  | *Tringa totanus* |
| Great Black-backed Gull |  | NEW |  |  |  |  |  |  |  |  |  |  |  |  |  |  |  | *Larus marinus* |
| Herring Gull | NEW |  |  |  |  |  |  |  |  |  |  |  |  |  |  |  |  | *Larus argentatus* |
| Lesser Black-backed Gull | NEW |  |  |  |  |  |  |  |  |  |  |  |  |  |  |  |  | *Larus fuscus* |
| Arctic Tern |  | NEW |  |  |  |  |  |  |  |  |  |  |  |  |  |  |  | *Sterna paradisaea* |
| Great Skua |  | NEW |  |  |  |  |  |  |  |  |  |  |  |  |  |  |  | *Stercorarius skua* |
| Arctic Skua |  | 27 |  |  |  |  |  |  |  |  |  |  |  |  |  |  |  | *Stercorarius parasiticus* |
| Ascension Frigatebird |  |  |  |  |  |  |  |  |  |  |  |  | 36 |  |  |  |  | *Fregata aquila* |
| Bittern |  |  |  |  |  |  |  |  |  |  | 5 |  |  |  |  |  |  | *Botaurus stellaris* |
| Little Bittern |  |  |  |  |  |  |  |  |  |  | 5 |  |  |  |  |  |  | *Botaurus minutus* |
| Grey Heron | 2 |  |  |  |  |  |  |  |  |  |  |  |  |  |  |  |  | *Ardea cinerea* |
| Purple Heron |  |  |  |  |  |  |  |  |  |  | 5 |  |  |  |  |  |  | *Ardea purpurea* |
| Osprey | NEW |  |  |  |  |  |  |  |  |  |  |  |  |  |  |  |  | *Pandion haliaetus* |
| Golden Eagle |  | NEW |  |  |  |  |  |  |  |  |  |  |  |  |  |  |  | *Aquila chrysaetos* |
| Sparrowhawk | 2 | 22 | NEW |  | NEW |  |  |  |  |  |  |  |  |  |  |  |  | *Accipiter nisus* |
| Goshawk | NEW |  |  |  |  |  |  |  |  |  |  |  |  |  |  |  |  | *Accipiter gentilis* |
| Hen Harrier |  | NEW |  |  |  |  |  |  |  |  |  |  |  |  |  |  |  | *Circus cyaneus* |
| Montagu's Harrier |  | 41 |  |  |  |  |  |  |  |  |  |  |  |  |  |  |  | *Circus pygargus* |
| Red Kite | NEW |  |  |  |  |  |  |  |  |  |  |  |  |  |  |  |  | *Milvus milvus* |
| Buzzard | 2 |  |  |  |  |  |  |  |  |  |  |  |  |  |  |  |  | *Buteo buteo* |
| Barn Owl | 2 | NEW |  |  | NEW |  |  |  |  |  |  |  |  |  |  |  |  | *Tyto alba* |
| Tawny Owl | 2 | 2 |  |  |  |  |  |  |  |  |  |  |  |  |  |  |  | *Strix aluco* |
| Little Owl | 2 | 30 |  |  |  |  |  |  |  |  |  |  |  |  |  |  |  | *Athene noctua* |
| Long-eared Owl | 2 | 22 | 13 |  |  |  |  |  |  |  |  |  |  |  |  |  |  | *Asio otus* |
| Short-eared Owl | 13 | 15 |  |  |  |  |  |  |  |  |  |  |  |  |  |  |  | *Asio flammeus* |
| Wryneck |  | NEW |  |  |  |  |  |  |  |  |  |  |  |  |  |  |  | *Jynx torquilla* |
| Great Spotted Woodpecker | 2 |  |  |  |  | NEW |  |  |  |  |  |  |  |  |  |  |  | *Dendrocopos major* |
| Green Woodpecker | 5 |  |  |  |  |  |  |  |  |  |  |  |  |  |  |  |  | *Picus viridis* |
| Kestrel | 2 | 13 |  |  |  |  |  |  |  |  |  |  |  |  |  |  |  | *Falco tinnunculus* |
| Red-footed Falcon |  | 13 |  |  |  |  |  |  |  |  |  |  |  |  |  |  |  | *Falco vespertinus* |
| Merlin | 25 | 38 |  |  |  |  |  |  |  |  |  |  |  |  |  |  |  | *Falco columbarius* |
| Hobby | NEW |  |  |  | NEW |  |  |  |  |  |  |  |  |  |  |  |  | *Falco subbuteo* |
| Peregrine | 2 |  |  |  |  |  |  |  |  |  |  |  |  |  |  |  |  | *Falco peregrinus* |
| Ring-necked Parakeet | 32 |  |  |  |  |  |  |  |  |  |  |  |  |  |  |  |  | *Psittacula krameri* |
| Red-backed Shrike | 2 | 13 |  |  |  |  |  |  |  |  |  |  |  |  |  |  |  | *Lanius collurio* |
| Jay | 2 |  |  |  |  |  |  |  |  |  |  |  |  |  |  |  |  | *Garrulus glandarius* |
| Magpie | 2 |  |  |  |  |  |  |  |  |  |  |  |  |  |  |  |  | *Pica pica* |
| Chough | NEW |  |  |  |  |  |  |  |  |  |  |  |  |  |  |  |  | *Pyrrhocorax pyrrhocorax* |
| Jackdaw | 2 |  |  |  |  |  |  |  |  |  |  |  |  |  |  |  |  | *Coloeus monedula* |
| Rook | 2 |  |  |  |  |  |  |  |  |  |  |  |  |  |  |  |  | *Corvus frugilegus* |
| Carrion Crow | 2 | 13 |  |  |  |  |  |  |  |  |  |  |  |  |  |  |  | *Corvus corone* |
| Hooded Crow | NEW | NEW |  |  |  |  |  |  |  |  |  |  |  |  |  |  |  | *Corvus cornix* |
| Coal Tit |  |  | NEW |  |  |  |  |  |  |  |  |  |  |  |  | NEW |  | *Periparus ater* |
| Willow Tit |  |  | NEW |  |  |  |  |  |  |  |  |  |  |  |  |  |  | *Poecile montanus* |
| Blue Tit | 19 | NEW | 41 |  |  |  |  |  |  |  |  |  |  |  |  |  |  | *Cyanistes caeruleus* |
| Great Tit | 2 | 23 | 26 |  |  |  |  |  |  |  |  |  |  |  |  |  |  | *Parus major* |
| Skylark |  | 13 | 29 |  |  |  |  |  |  |  |  |  |  |  |  |  |  | *Alauda arvensis* |
| Sand Martin |  | 6 | 6 | 7 |  | 1 |  |  |  |  |  |  |  |  |  |  |  | *Riparia riparia* |
| Swallow | 8 | 13 | 6 | 9 | 6 | 10 |  |  |  |  |  |  |  |  |  |  |  | *Hirundo rustica* |
| House Martin | 1 |  | 2 |  | 2 | 11 |  |  |  |  |  |  |  |  |  |  |  | *Delichon urbicum* |
| Cetti's Warbler |  |  | NEW |  |  |  |  |  |  |  |  |  |  |  |  |  |  | *Cettia cetti* |
| Long-tailed Tit | 12 |  | 8 |  |  |  |  |  |  |  |  |  |  |  |  |  |  | *Aegithalos caudatus* |
| Willow Warbler | 13 | 13 | 14 |  |  |  |  |  |  |  |  |  |  |  |  |  |  | *Phylloscopus trochilus* |
| Chiffchaff |  |  | 5 |  |  |  |  |  |  |  |  |  |  |  |  |  |  | *Phylloscopus collybita* |
| Sedge Warbler | NEW | 15 | 13 |  |  |  |  |  |  |  | NEW |  |  |  |  |  |  | *Acrocephalus schoenobaenus* |
| Paddyfield Warbler |  |  |  | 7 |  |  |  |  |  |  |  |  |  |  |  |  |  | *Acrocephalus agricola* |
| Reed Warbler | 16 |  | 13 |  |  |  |  |  |  |  |  |  |  |  |  |  |  | *Acrocephalus scirpaceus* |
| Icterine Warbler |  | 13 |  |  |  |  |  |  |  |  |  |  |  |  |  |  |  | *Hippolais icterina* |
| Grasshopper Warbler | NEW | NEW |  |  |  |  |  |  |  |  |  |  |  |  |  |  |  | *Locustella naevia* |
| Blackcap | NEW | NEW | 13 |  |  |  |  |  |  |  |  |  |  |  |  |  |  | *Sylvia atricapilla* |
| Garden Warbler | NEW | 18 | 17 |  |  |  |  |  |  |  |  |  |  |  |  |  |  | *Sylvia borin* |
| Barred Warbler |  |  | 13 |  |  |  |  |  |  |  |  |  |  |  |  |  |  | *Curruca nisoria* |
| Lesser Whitethroat | 2 |  | 19 |  |  |  |  |  |  |  |  |  |  |  |  |  |  | *Curruca curruca* |
| Whitethroat | 2 | 13 | 17 |  |  |  |  |  |  | 20 |  |  |  |  |  |  |  | *Curruca communis* |
| Dartford Warbler |  | 13 | 21 |  |  |  |  |  |  |  |  |  |  |  |  |  |  | *Curruca undata* |
| Firecrest |  |  | NEW |  |  |  |  |  |  |  |  |  |  |  |  |  |  | *Regulus ignicapilla* |
| Goldcrest |  | 22 | 13 |  |  |  |  |  |  |  |  |  |  |  |  | NEW |  | *Regulus regulus* |
| Wren |  | 13 | 23 |  |  |  |  |  |  |  |  |  |  |  |  |  |  | *Troglodytes troglodytes* |
| Nuthatch | 41 |  | 41 |  |  |  |  |  |  |  |  |  |  |  |  |  |  | *Sitta europaea* |
| Treecreeper |  |  | 23 |  |  |  |  |  |  |  |  |  |  |  |  |  |  | *Certhia familiaris* |
| Starling | 2 | 2 | 14 |  |  |  |  |  |  |  |  |  |  |  |  |  |  | *Sturnus vulgaris* |
| White's Thrush |  | 13 |  |  |  |  |  |  |  |  |  |  |  |  |  |  |  | *Zoothera aurea* |
| Ring Ouzel |  | 24 |  |  |  |  |  |  |  |  |  |  |  |  |  |  |  | *Turdus torquatus* |
| Blackbird | 2 | 22 | NEW |  |  |  |  |  |  |  |  |  |  |  |  |  |  | *Turdus merula* |
| Redwing | 13 | 25 |  |  |  |  |  |  |  |  |  |  |  |  |  | NEW |  | *Turdus iliacus* |
| Song Thrush | 2 | 13 | NEW |  |  |  |  |  |  |  |  |  |  |  |  |  |  | *Turdus philomelos* |
| Mistle Thrush | 41 |  |  |  |  |  |  |  |  |  |  |  |  |  |  |  |  | *Turdus viscivorus* |
| Spotted Flycatcher | 41 | 13 | 21 |  |  |  |  |  |  |  |  |  |  |  |  |  |  | *Muscicapa striata* |
| Robin | 2 | 23 | 26 |  |  |  |  |  |  |  |  |  |  |  |  |  |  | *Erithacus rubecula* |
| Bluethroat |  | 13 |  |  |  |  |  |  |  |  |  |  |  |  |  |  |  | *Luscinia svecica* |
| Pied Flycatcher | NEW | 13 |  |  |  |  |  |  |  |  |  |  |  |  |  |  |  | *Ficedula hypoleuca* |
| Redstart | NEW | 13 | NEW |  |  |  |  |  |  |  |  |  |  |  |  |  |  | *Phoenicurus phoenicurus* |
| Whinchat |  | 13 |  |  |  |  |  |  |  |  |  |  |  |  |  |  |  | *Saxicola rubetra* |
| Stonechat | 21 | NEW | 13 |  |  |  |  |  |  |  |  |  |  |  |  |  |  | *Saxicola rubicola* |
| Wheatear | 2 | 27 | 2 |  |  |  |  |  |  |  |  |  |  |  |  |  |  | *Oenanthe oenanthe* |
| House Sparrow | 2 | 38 | 13 |  |  |  |  |  |  |  |  |  |  |  |  |  |  | *Passer domesticus* |
| Tree Sparrow | 9 | 23 | 23 |  |  |  |  |  |  |  |  |  |  |  |  |  |  | *Passer montanus* |
| Dunnock | 2 | 13 | 17 |  |  |  |  |  |  |  |  |  |  |  |  |  |  | *Prunella modularis* |
| Yellow Wagtail |  | 13 | 13 |  |  |  |  |  |  |  |  |  |  |  |  |  |  | *Motacilla flava* |
| Citrine Wagtail |  | 25 |  |  |  |  |  |  |  |  |  |  |  |  |  |  |  | *Motacilla citreola* |
| Pied Wagtail | 23 | 13 | 14 |  |  |  |  |  |  |  |  |  |  |  |  |  |  | *Motacilla alba* |
| Meadow Pipit | NEW | 27 | 2 |  |  |  |  |  |  |  |  |  |  |  |  |  |  | *Anthus pratensis* |
| Tree Pipit | NEW | 22 | 2 |  |  |  |  |  |  |  |  | 28 |  |  |  |  |  | *Anthus trivialis* |
| Rock Pipit | 29 | 27 | 29 |  |  |  |  |  |  |  |  |  |  |  |  |  |  | *Anthus petrosus* |
| Chaffinch | 41 | NEW | 2 |  |  |  |  |  |  |  |  |  |  |  |  |  |  | *Fringilla coelebs* |
| Bullfinch | 13 | 23 | NEW |  |  |  |  |  |  |  |  |  |  |  |  |  |  | *Pyrrhula pyrrhula* |
| Greenfinch | 19 | NEW | 19 |  |  |  |  |  |  |  |  |  |  |  |  |  |  | *Chloris chloris* |
| Twite | 25 | 27 |  |  |  |  |  |  |  |  |  |  |  |  |  |  |  | *Linaria flavirostris* |
| Linnet | 19 | 23 | 19 |  |  |  |  |  |  |  |  |  |  |  |  |  |  | *Linaria cannabina* |
| Redpoll |  | 30 | NEW |  |  |  |  |  |  |  |  |  |  |  |  |  |  | *Acanthis flammea* |
| Crossbill | NEW | 13 |  |  |  |  |  |  |  |  |  |  |  |  |  |  |  | *Loxia curvirostra* |
| Goldfinch | NEW | NEW | 21 |  |  |  |  |  |  |  |  |  |  |  |  |  |  | *Carduelis carduelis* |
| Siskin | NEW | NEW | NEW |  |  |  |  |  |  |  |  |  |  |  |  |  |  | *Spinus spinus* |
| Corn Bunting | 21 |  |  |  |  |  |  |  |  |  |  |  |  |  |  |  |  | *Emberiza calandra* |
| Yellowhammer | NEW | 13 | 26 |  |  |  |  |  |  |  |  |  |  |  |  |  |  | *Emberiza citrinella* |
| Reed Bunting | NEW | 31 | 13 |  |  |  |  |  |  |  |  |  |  |  |  |  |  | *Emberiza schoeniclus* |
| Sheep |  |  |  |  |  |  |  |  |  |  |  |  |  |  |  |  | 28 | *Ovis aries* |
| Reindeer |  |  |  |  |  |  |  |  |  |  |  |  |  |  |  | 37 |  | *Rangifer tarandus* |
| Fallow Deer |  |  |  |  |  |  |  |  |  |  |  |  |  |  |  | 3 |  | *Dama dama* |
| Roe Deer |  |  |  |  |  |  |  |  |  |  |  |  |  |  |  | 3 |  | *Capreolus capreolus* |
| Sika Deer |  |  |  |  |  |  |  |  |  |  |  |  |  |  |  | 28 |  | *Cervus nippon* |
| Red Deer |  |  |  |  |  |  |  |  |  |  |  |  |  |  |  | 3 |  | *Cervus elaphus* |
| Cattle |  |  |  |  |  |  |  |  |  |  |  |  |  |  | 4 |  |  | *Bos taurus* |
| Horse |  |  |  |  |  |  |  |  |  |  |  |  |  |  | 4 | NEW |  | *Equus ferus caballus* |
| Badger |  |  |  |  |  |  |  |  |  |  |  |  |  |  |  | 28 |  | *Meles Meles* |
| Dog |  |  |  |  |  |  |  |  |  |  |  |  |  |  | 4 | 28 |  | *Canis familiaris* |
| Human | 21 | NEW | 21 |  | 2 |  |  |  |  |  |  |  |  |  | 28 | 28 |  | *Homo sapiens* |
| ACCIDENTAL IMPORTS |  |  |  |  |  |  |  |  |  |  |  |  |  |  |  |  |  |  |
| Pekin Robin/Red-billed Leiothrix |  |  |  |  |  |  |  |  | 28 |  |  |  |  |  |  |  |  | *Leiothrix lutea* |
| Ring-necked Parakeet |  |  |  |  |  |  |  |  | 28 |  |  |  |  |  |  |  |  | *Psittiacula krameri* |
| Cheetah |  |  |  |  |  |  |  |  |  |  |  |  |  | 39 |  |  |  | *Acinonyx jubatus* |

References

1. Thompson GB. Contributions toward a study of the ectoparasites of British birds and mammals.—No. 1. Ann Mag Nat Hist. 1953;6(66):401–25.

2. Thompson GB. V.—Contributions toward a study of the ectoparasites of British birds and mammals.—No. 2. Ann Mag Nat Hist [Internet]. 1954 Jan 3;7(73):17–39. Available from: https://www.tandfonline.com/doi/full/10.1080/00222935508651820

3. Thompson GB. LIX.—Contributions toward a study of the ectoparasites of British birds and mammals.—No. 3. Ann Mag Nat Hist [Internet]. 1954 Jun 10;7(78):438–47. Available from: https://www.tandfonline.com/doi/full/10.1080/00222935408656061

4. Thompson GB. II.—Contributions toward a study of the ectoparasites of British birds and mammals—No. 4. Ann Mag Nat Hist [Internet]. 1955 Jan 10;8(85):25–35. Available from: https://www.tandfonline.com/doi/full/10.1080/00222935508651820

5. Thompson GB. LXXXIX.—Contributions toward a study of the Ectoparasites of British birds and Mammals.—No. 5. Ann Mag Nat Hist [Internet]. 1955 Oct 4;8(94):724–30. Available from: https://doi.org/10.1080/00222935508655693

6. Thompson GB, Beaumont HE. Some interesting Records of Flat-flies (Diptera: Hippoboscidae) from Hirundinidae & Apodidae. In: The Naturalist. https://www.biodiversitylibrary.org/page/50236244#page/7/mode/1up; 1968. p. 111–4.

7. Wawman DC. Ornithomya biloba , Pseudolynchia garzettae and Pseudolynchia canariensis (Diptera : Hippoboscidae): three new United Kingdom colonists and potential disease vectors. Med Vet Entomol [Internet]. 2024;38(2):160–71. Available from: https://resjournals.onlinelibrary.wiley.com/doi/full/10.1111/mve.12703

8. Beaumont HE. A review of the records of Yorkshire Hippoboscidae (Diptera). Naturalist. 1965;99–101.

9. Lloyd-Evans L. Bird Parasites. Rye Meads Ringing Gr Rep. 1967;4:14–21.

10. Waterson J. IX. - Notes on Some Ectoparasites in the Museum, Perth. Trans - Perthsh Soc Nat Sci. 1910;48–9.

11. Chandler PJ. Ethel Katharine Pearce (1856-1940) and her contribution to dipterology. Dipterists Dig Second Ser. 2009;16(2):117–46.

12. Denton ML. Ectoparasites. Birds Huddersf. 1978;59.

13. Hill DS. A STUDY OF THE DISTRIBUTION AND HOST PREFERENCES OF THREE SPECIES OF ORNITHOMYIA (DIPTERA: HIPPOBOSCIDAE) IN THE BRITISH ISLES. 1962 Apr 2;37(4–6):37–48. Available from: http://doi.wiley.com/10.1111/j.1365-3032.1962.tb00286.x

14. Stansfield G. Flat-flies and Fleas from Skokholm Birds, 1955. Skokholm Bird Obs Rep. 1955;26.

15. Sellers RM, Redgate ND. Some recent records of the grouse louse-fly, Ornithomya chloropus Bergroth (Diptera: Hippoboscidae) from Caithness. Entomol Gaz. 1992;43(4):274.

16. Emley DW. Staffordshire Flies, a provisional list [Internet]. 1992. Available from: https://www.google.com/url?sa=t&rct=j&q=&esrc=s&source=web&cd=&ved=2ahUKEwjPxK-sx57uAhUfQxUIHTQOATwQFjAAegQIBhAC&url=http%3A%2F%2Fwww.staffs-ecology.org.uk%2Fhtml2015%2Fimages%2F6%2F67%2FSER015_Staffordshire_Flies_-_A_Provisional_List.pdf&usg=AOvVaw0cBw-0

17. Thompson GB. Ectoparasites. Gibraltar Point Rep. 1952;30.

18. Edwards R. Report on Bird Ectoparasites. Fair Isle Bird Obs Annu Rep 1950 [Internet]. 1951;162:21. Available from: http://www.fairislebirdobs.co.uk/annual_reports.html

19. Ash JS, Monk JF. A collection of Ornithomyia spp. (Dipt., Hippoboscidae) from Oxfordshire. Entomol Mon Mag. 1959;xcv(10th September):80–1.

20. Thompson GB. Ornithophila metallica (Schiner) (Diptera: Hippoboscidae) A species of flat-fly new to the British List. Bardsey Obs Rep 1967. 1968;46–7.

21. Ash JS. Some records of bird and mammal ectoparasites. Entomol Mon Mag. 1955;xc1:64–5.

22. Edwards AR. Flatflies taken in the Laboratory during 1951. Fair Isle Bird Obs Annu Rep 1951 [Internet]. 1952;(37–38). Available from: http://www.fairislebirdobs.co.uk/annual_reports.html

23. Denton ML. Ectoparasites. Annu Rep 1974 Huddersf Birdwatchers’ Club. 1974;9–12.

24. Denton ML. Ectoparasites. Status Birds Hudderf Area 1959-1974, Annu Rep 1975. 1975;9–10.

25. Corbet GC. Research on Flat-flies at Fair Isle in 1954. Fair Isle Bird Obs Bull [Internet]. 1955;2(7):313–7. Available from: http://www.fairislebirdobs.co.uk/annual_reports.html

26. Curtis J. Ornithomyia fringillina. Br Entomol. 1836;13:582–3.

27. Williamson K. Bird Parasites. Fair Isle Bird Obs Annu Report, 1949 [Internet]. 1950;22. Available from: http://www.fairislebirdobs.co.uk/annual_reports.html

28. Hutson AM. Keds, Flat-flies and Bat-Flies: Diptera, Hippoboscidae and Nycteribiidae. Handbooks Identif Br Insects [Internet]. 1984;10(7):40. Available from: https://www.royensoc.co.uk/sites/default/files/Vol10_Part07_Hutson.pdf

29. Stansfield G. Collection of Flat-flies on Skokholm Island. Skokholm Bird Obs Rep. 1954;19–22.

30. Denton ML. Ectoparasites. Huddersf BIRDWATCHERS ’ CLUB Annu Rep 1977 [Internet]. 1977;42. Available from: http://www.huddersfieldbirdwatchersclub.co.uk/annual-reports/4559882266

31. Cutts DB. Some recent records of Hippoboscidae (Diptera), including Stenepteryx hirundinis L. from Swallow in V.C. 61. Bull Hull Nat Hist Soc. 1971;3–7.

32. Harris DD. First record from a parakeet of Ornithomyia avicularia (Linnaeus, 1758) (Diptera, Hippoboscidae). Dipterists Dig [Internet]. 2009;16(2):101. Available from: https://www.dipterists.org.uk/digest

33. Theobald F V. Parasitic diseases of poultry [Internet]. Parasitic diseases of poultry. London: Gurney; 1896. 34–36 p. Available from: https://www.biodiversitylibrary.org/bibliography/43848

34. Palmer CJ. Pseudolychnia garzettae Rondani (Dipt., Hippoboscidae), an unrecognized addition to the British List. Entomol Mon Mag. 1987;123:234.

35. White G. Letter 21 to Daines Barrington, Selborne, September 28th 1774. In: The Natural History of Selborne. 1789.

36. Graham J, Williams I, Kinnear N, Stpehen AC, Wynne-Edwards VC. Magnificent Frigate Bird in Tiree, Inner Hebrides; A New British Bird. Br Birds. 1954;47(2):58–9.

37. Kettle DS, Utsi MNP. Hypoderma diana (Diptera, Oestridae) and Lipoptena cervi (Diptera, Hippoboscidae) as parasites of reindeer (Rangifer tarandus) in Scotland with notes on the second-stage larva of Hypoderma diana. Parasitology. 1955;45(1–2):116–20.

38. Williamson K. Fair Isle Bird Observatory, First Report 1948. Scottish Nat [Internet]. 1949 Dec;19(1):19–142. Available from: http://www.fairislebirdobs.co.uk/annual_reports.html

39. O’Connor JP, Sleeman DP. A Review of the Irish Hippoboscidae (Insecta:Diptera). Irish Nat J. 1987;22(6 (April)):236–9.

40. Corbet GB. The Life-History and Host-Relations of a Hippoboscid Fly Ornithomyia fringillina Curtis. J Anim Ecol [Internet]. 1956 Nov;25(2):403. Available from: https://www.jstor.org/stable/1934?origin=crossref

41. Ash J. Records of Hippoboscidae (Dipt.) from Berkshire and Co. Durham in 1950, with notes on their bionomics. Entomol Mon Mag. 1952;lxxxviii:25–30.

42. Denton ML. Ectoparasites. Birds Huddersf 1979. 1979;50.
