# Supplementary material for: Citizen science reveals host‐switching in louse flies and keds (Diptera: Hippoboscidae) during a period of anthropogenic change
Source: Med Vet Entomol. 2025 Nov 1;40(2):305–22. doi: 10.1111/mve.70029 (PMC13140014; doi:10.1111/mve.70029)
Supplement: Supplementary file 4 — Data S4. A table of the network metrics calculated by the bipartite package for all species of Hippoboscidae in the current study: (a) species metrics, (b) network metrics. [file MVE-40-305-s004.docx]

**S4. Network metrics for all species from the current study**

1. Species metrics

|  | ***O. avicularia*** | ***O. biloba*** | ***O. chloropus*** | ***O. fringillina*** | ***C. pallida*** | ***S. hirundinis*** |
| --- | --- | --- | --- | --- | --- | --- |
| degree | 74 | 2 | 64 | 47 | 5 | 5 |
| normalised degree | 0.637931034 | 0.017241379 | 0.551724138 | 0.405172414 | 0.043103448 | 0.043103448 |
| species strength | 44.58614419 | 0.837092732 | 34.94085136 | 24.60112686 | 1.228254546 | 1.944106069 |
| interaction push pull | 0.589001948 | -0.081453634 | 0.530325803 | 0.502151635 | 0.045650909 | 0.188821214 |
| nestedrank | 0 | 0.545454545 | 0.090909091 | 0.181818182 | 0.454545455 | 0.363636364 |
| PDI | 0.965716538 | 0.999613527 | 0.970885093 | 0.947891963 | 0.999248524 | 0.998269112 |
| resource range | 0.365217391 | 0.991304348 | 0.452173913 | 0.6 | 0.965217391 | 0.965217391 |
| species specificity index | 0.255011782 | 0.958022234 | 0.297781907 | 0.240189123 | 0.920794233 | 0.845365189 |
| PSI | 0.763759715 | 0.757905402 | 0.691391516 | 0.653482991 | 0.912538744 | 0.951856639 |
| node specialisation index NSI | 1.1 | 1.7 | 1.3 | 1.3 | 1.7 | 1.6 |
| betweenness | 0.550438596 | 0 | 0.103070175 | 0.103070175 | 0 | 0.006578947 |
| weighted betweenness | 0.459349593 | 0 | 0.113821138 | 0.134146341 | 0 | 0.146341463 |
| closeness | 0.125 | 0.089912281 | 0.111842105 | 0.111842105 | 0.089912281 | 0.096491228 |
| weighted closeness | 0.003414942 | 0.003190928 | 0.003400286 | 0.003408246 | 0.003030389 | 0.003170291 |
| Fisher alpha | NA | NA | NA | NA | NA | NA |
| partner diversity | 3.215848312 | 0.175975125 | 2.983992958 | 3.027604097 | 0.379240079 | 0.528876185 |
| effective partners | 24.92442662 | 1.192408397 | 19.76658643 | 20.64770338 | 1.46117379 | 1.697024096 |
| proportional generality | 0.540122337 | 0.025839969 | 0.428349868 | 0.447444027 | 0.031664223 | 0.036775194 |
| proportional similarity | 0.568767004 | 0.024270655 | 0.482515997 | 0.418222766 | 0.083858009 | 0.082523326 |
| d | 0.588972467 | 0.915978748 | 0.558245396 | 0.620026734 | 0.91906373 | 0.963452836 |

|  | ***P. canariensis*** | ***P. garzettae*** | ***I. minor*** | ***H. equina*** | ***L. cervi*** | ***M. ovinus*** |
| --- | --- | --- | --- | --- | --- | --- |
| degree | 1 | 1 | 1 | 1 | 8 | 1 |
| normalised degree | 0.00862069 | 0.00862069 | 0.00862069 | 0.00862069 | 0.068965517 | 0.00862069 |
| species strength | 0.857142857 | 0.6 | 0.083333333 | 0.666666667 | 4.655281385 | 1 |
| interaction push pull | -0.142857143 | -0.4 | -0.916666667 | -0.333333333 | 0.456910173 | 0 |
| nestedrank | 0.727272727 | 0.909090909 | 1 | 0.818181818 | 0.272727273 | 0.636363636 |
| PDI | 1 | 1 | 1 | 1 | 0.987198068 | 1 |
| resource range | 1 | 1 | 1 | 1 | 0.939130435 | 1 |
| species specificity index | 1 | 1 | 1 | 1 | 0.508277217 | 1 |
| PSI | 0.857142857 | 0.6 | 0.083333333 | 0.666666667 | 0.935520697 | 1 |
| node specialisation index NSI | 2 | 2 | 1.8 | 2.5 | 1.6 | NA |
| betweenness | 0 | 0 | 0 | 0 | 0.236842105 | 0 |
| weighted betweenness | 0 | 0 | 0 | 0 | 0.146341463 | 0 |
| closeness | 0.070175439 | 0.070175439 | 0.083333333 | 0.059210526 | 0.092105263 | 0 |
| weighted closeness | 0.002647297 | 0.001529727 | 0.000649137 | 0.001674691 | 0.002920682 | 0 |
| Fisher alpha | NA | NA | NA | NA | NA | NA |
| partner diversity | 0 | 0 | 0 | 0 | 1.519280007 | 0 |
| effective partners | 1 | 1 | 1 | 1 | 4.56893441 | 1 |
| proportional generality | 0.021670402 | 0.021670402 | 0.021670402 | 0.021670402 | 0.099010644 | 0.021670402 |
| proportional similarity | 0.001716107 | 0.001225791 | 0.002941898 | 0.001470949 | 0.036487242 | 0.013483697 |
| d | 0.971652831 | 0.909317326 | 0.581491958 | 0.927157521 | 0.965575898 | 1 |

1. Network metrics

| connectance | 0.062056738 |
| --- | --- |
| web asymmetry | -0.91836735 |
| links per species | 0.714285714 |
| number of compartments | 2 |
| compartment diversity | 1.487469234 |
| cluster coefficient | 0.012411348 |
| modularity Q | 0.541110205 |
| nestedness | 0.328057014 |
| NODF | 8.027472217 |
| weighted nestedness | 0.696069989 |
| weighted NODF | 4.226329785 |
| interaction strength asymmetry | -0.40715892 |
| specialisation asymmetry | 0.388362937 |
| linkage density | 10.66744442 |
| weighted connectance | 0.036283825 |
| Fisher alpha | 46.90793502 |
| Shannon diversity | 4.280137048 |
| interaction evenness | 0.526668532 |
| Alatalo interaction evenness | 0.56546745 |
| H2 | 0.698574421 |
| Number of species HL | 12 |
| Number of species LL | 282 |
| Mean number of shared partners HL | 1.984848485 |
| Mean number of shared partners LL | 0.147573257 |
| Cluster coefficient HL | 0.199396146 |
| Cluster coefficient LL | 0.203031789 |
| Weighted cluster coefficient HL | 0.311972002 |
| Weighted cluster coefficient LL | 0.902750337 |
| Niche overlap HL | 0.013151516 |
| Niche overlap LL | 0.360204403 |
| Togetherness HL | 0.025680401 |
| Togetherness LL | 0.038647615 |
| C score HL | 0.769453565 |
| C score LL | 0.35483654 |
| V ratio HL | 48.21679688 |
| V ratio LL | 1.881055034 |
| Discrepancy HL | 150 |
| Discrepancy LL | 71 |
| Extinction slope HL | 0.319090285 |
| Extinction slope LL | 0.525437841 |
| Robustness HL | 0.221368562 |
| Robustness LL | 0.298146446 |
| Functional complementarity HL | 1940.015143 |
| Functional complementarity LL | 2519.633151 |
| Partner diversity HL | 2.763589388 |
| Partner diversity LL | 0.448329122 |
| Generality HL | 19.65590981 |
| Vulnerability LL | 1.678979035 |
