# Supplementary material for: Citizen science reveals host‐switching in louse flies and keds (Diptera: Hippoboscidae) during a period of anthropogenic change
Source: Med Vet Entomol. 2025 Nov 1;40(2):305–22. doi: 10.1111/mve.70029 (PMC13140014; doi:10.1111/mve.70029)
Supplement: Supplementary file 5 — Data S5. A table comparing the network metrics calculated by the bipartite package the three generalist species in the genus Ornithomya (O. avicularia, O. chloropus and O. fringillina) from the 1960s study (Hill, 1962a) and the current study: (a) species metrics, (b) network metrics. [file MVE-40-305-s005.docx]

**S5. A comparison of the network and species metrics from Hill (1962) with the current study for the three generalist Ornithomya species**

1. Species Metrics

|  | **Hill, 1962** | | | **Current study** | | |
| --- | --- | --- | --- | --- | --- | --- |
|  | ***O. avicularia*** | ***O. chloropus*** | ***O. fringillina*** | ***O. avicularia*** | ***O. chloropus*** | ***O. fringillina*** |
| degree | 43 | 56 | 25 | 74 | 64 | 47 |
| normalised degree | 0.483146067 | 0.629213483 | 0.280898876 | 0.678899083 | 0.587155963 | 0.431192661 |
| species strength | 30.15866039 | 42.27262843 | 16.56871117 | 47.30966764 | 36.69991057 | 24.99042179 |
| interaction push pull | 0.678108381 | 0.737011222 | 0.622748447 | 0.625806319 | 0.557811103 | 0.510434506 |
| nestedrank | 0.5 | 0 | 1 | 0 | 0.5 | 1 |
| PDI | 0.972348485 | 0.964022727 | 0.992272727 | 0.963494461 | 0.968998016 | 0.94451459 |
| resource.range | 0.522727273 | 0.375 | 0.727272727 | 0.324074074 | 0.416666667 | 0.574074074 |
| species specificity index | 0.437585506 | 0.416682865 | 0.620386913 | 0.253985425 | 0.296925685 | 0.239090571 |
| PSI | 0.84244066 | 0.930199012 | 0.910756303 | 0.769052816 | 0.695710484 | 0.656085624 |
| node specialisation index NSI | 1 | 1 | 1 | 1 | 1 | 1 |
| betweenness | 0 | 0 | 0 | 0 | 0 | 0 |
| weighted betweenness | 0 | 1 | 0 | 0 | 0 | 0 |
| closeness | 0.333333333 | 0.333333333 | 0.333333333 | 0.333333333 | 0.333333333 | 0.333333333 |
| weighted closeness | 0.253978868 | 0.46213907 | 0.482473842 | 0.605720071 | 0.39969812 | 0.471838586 |
| Fisher alpha | 9.069509294 | 10.59316743 | 5.824961963 | 15.9467077 | 15.36664586 | 10.4658841 |
| partner diversity | 2.119669196 | 1.985464436 | 1.522202484 | 3.215848312 | 2.983992958 | 3.027604097 |
| effective partners | 8.328381969 | 7.28242882 | 4.582306544 | 24.92442662 | 19.76658643 | 20.64770338 |
| proportional generality | 0.541491216 | 0.473485876 | 0.297930469 | 0.626289828 | 0.49668593 | 0.518826242 |
| proportional similarity | 0.426183914 | 0.641212223 | 0.141755961 | 0.63214889 | 0.518457245 | 0.470453937 |
| d | 0.747958646 | 0.821632863 | 0.939128446 | 0.527611754 | 0.522059746 | 0.584157452 |

1. Network Metrics, calculated using the same species matrix for both studies.

|  | **Hill, 1962** | **Current Study** |
| --- | --- | --- |
| connectance | 0.146572104 | 0.218676123 |
| web asymmetry | -0.978947368 | -0.978947368 |
| links per species | 0.435087719 | 0.649122807 |
| number of compartments | 1 | 1 |
| compartment diversity | NA | NA |
| cluster coefficient | 0.15248227 | 0.226950355 |
| modularity Q | 0.479253993 | 0.436534141 |
| nestedness | 4.919740816 | 10.0260877 |
| NODF | 3.660295146 | 8.035314201 |
| weighted nestedness | 0.239844204 | 0.165464041 |
| weighted NODF | 1.557977547 | 4.294901663 |
|  | **Hill, 1962** | **Current Study** |
| interaction strength asymmetry | -0.544433976 | -0.468035443 |
| specialisation asymmetry | 0.48201482 | 0.541496903 |
| linkage density | 4.238152126 | 12.06025872 |
| weighted connectance | 0.014870709 | 0.042316697 |
| Fisher alpha | 25.01211869 | 41.5232781 |
| Shannon diversity | 2.89353342 | 4.1651485 |
| interaction evenness | 0.429274551 | 0.61792694 |
| Alatalo interaction evenness | 0.621777808 | 0.557740323 |
| H2 | 0.824823549 | 0.543455919 |
| number of species HL | 3 | 3 |
| number of species LL | 282 | 282 |
| mean number of shared partners HL | 13.66666667 | 33.33333333 |
| mean number of shared partners LL | 0.069230963 | 0.146336539 |
| cluster coefficient HL | 0.17207364 | 0.227621835 |
| cluster coefficient LL | 0.547787527 | 0.798188508 |
| weighted cluster coefficient HL | 0.091204409 | 0.31147565 |
| weighted cluster coefficient LL | 1 | 1 |
| niche overlap HL | 0.073456872 | 0.240820641 |
| niche overlap LL | 0.415990723 | 0.444655954 |
| togetherness HL | 0.229193005 | 0.456852064 |
| togetherness LL | 0.033656394 | 0.033038035 |
| C score HL | 0.403565891 | 0.190309325 |
| C score LL | 0.468410226 | 0.473389356 |
| V ratio HL | 8.759036145 | 6.576470588 |
| V ratio LL | 1.455003747 | 1.840723959 |
| discrepancy HL | 133 | 129 |
| discrepancy LL | 45 | 49 |
| extinction slope HL | 0.271424641 | 0.395856508 |
| extinction slope LL | 0.556650332 | 0.732943629 |
| robustness HL | 0.088833727 | 0.132927502 |
| robustness LL | 0.307458194 | 0.38040196 |
| functional complementarity HL | 2373.055928 | 1253.841098 |
| functional complementarity LL | 2366.931756 | 1989.0383 |
| partner diversity HL | 1.969517898 | 3.102749946 |
| partner diversity LL | 0.16043579 | 0.481358158 |
| generality HL | 7.266374585 | 22.38518181 |
| vulnerability LL | 1.209929668 | 1.735335617 |
